# Tuberculosis in otherwise healthy adults with inherited TNF deficiency

**DOI:** 10.1038/s41586-024-07866-3

**Published:** 2024-08-28

**Authors:** Andrés A. Arias, Anna-Lena Neehus, Masato Ogishi, Vincent Meynier, Adam Krebs, Tomi Lazarov, Angela M. Lee, Carlos A. Arango-Franco, Rui Yang, Julio Orrego, Melissa Corcini Berndt, Julian Rojas, Hailun Li, Darawan Rinchai, Lucia Erazo-Borrás, Ji Eun Han, Bethany Pillay, Khoren Ponsin, Matthieu Chaldebas, Quentin Philippot, Jonathan Bohlen, Jérémie Rosain, Tom Le Voyer, Till Janotte, Krishnajina Amarajeeva, Camille Soudée, Marion Brollo, Katja Wiegmann, Quentin Marquant, Yoann Seeleuthner, Danyel Lee, Candice Lainé, Doreen Kloos, Rasheed Bailey, Paul Bastard, Narelle Keating, Franck Rapaport, Taushif Khan, Marcela Moncada-Vélez, María Camila Carmona, Catalina Obando, Jesús Alvarez, Juan Carlos Cataño, Larry Luber Martínez-Rosado, Juan P. Sanchez, Manuela Tejada-Giraldo, Anne-Sophie L’Honneur, María L. Agudelo, Lizet J. Perez-Zapata, Diana M. Arboleda, Juan Fernando Alzate, Felipe Cabarcas, Alejandra Zuluaga, Simon J. Pelham, Armin Ensser, Monika Schmidt, Margarita M. Velásquez-Lopera, Emmanuelle Jouanguy, Anne Puel, Martin Krönke, Stefano Ghirardello, Alessandro Borghesi, Susanta Pahari, Bertrand Boisson, Stefania Pittaluga, Cindy S. Ma, Jean-François Emile, Luigi D. Notarangelo, Stuart G. Tangye, Nico Marr, Nico Lachmann, Hélène Salvator, Larry S. Schlesinger, Peng Zhang, Michael S. Glickman, Carl F. Nathan, Frédéric Geissmann, Laurent Abel, José Luis Franco, Jacinta Bustamante, Jean-Laurent Casanova, Stéphanie Boisson-Dupuis

**Affiliations:** 1 Inborn Errors of Immunity Group, Department of Microbiology and Parasitology, School of Medicine, University of Antioquia UdeA, Medellín, Colombia; 2 School of Microbiology, University of Antioquia UdeA, Medellín, Colombia; 3 St Giles Laboratory of Human Genetics of Infectious Diseases, Rockefeller Branch, Rockefeller University, New York, NY USA; 4 Laboratory of Human Genetics of Infectious Diseases, Necker Branch, INSERM U1163, Paris, France; 5 Paris Cité University, Imagine Institute, Paris, France; 6 Immunology Program, Sloan Kettering Institute, Memorial Sloan Kettering Cancer Center, New York, NY USA; 7 Immunology and Microbial Pathogenesis Program, Weill Cornell Medicine, New York, NY USA; 8 Department of Microbiology & Immunology, Weill Cornell Medicine, New York, NY USA; 9 Garvan Institute of Medical Research, Darlinghurst, New South Wales Australia; 10 School of Clinical Medicine, Faculty of Medicine and Health, UNSW Sydney, Kensington, New South Wales Australia; 11 Study Center for Primary Immunodeficiencies, Necker Hospital for Sick Children, Assistance publique–Hôpitaux de Paris (AP-HP), Paris, France; 12 Clinical Immunology Department, AP-HP, Saint-Louis Hospital, Paris, France; 13 Lab VIM Suresnes, UMR 0892, Paris Saclay University, INRAe UVSQ, Suresnes, France; 14 Institute for Medical Microbiology, Immunology and Hygiene, Faculty of Medicine and University Hospital Cologne, University of Cologne, Cologne, Germany; 15 Department of Pediatric Pneumology, Allergology and Neonatology, Hannover Medical School, Hannover, Germany; 16 REBIRTH—Research Center for Translational Regenerative Medicine, Hannover, Germany; 17 Pediatric Immunology-Hematology and Rheumatology Unit, Necker Hospital for Sick Children, AP-HP, Paris, France; 18 Walter and Eliza Hall Institute of Medical Research, Melbourne, Victoria Australia; 19 Department of Medical Biology, University of Melbourne, Melbourne, Victoria Australia; 20 The Jackson Laboratory, Farmington, CT USA; 21 Infectious Diseases Section, Department of Internal Medicine, School of Medicine, University of Antioquia UdeA, Medellín, Colombia; 22 Latin American Research Team in Infectiology and Public Health (ELISAP), La Maria Hospital, Medellín, Colombia; 23 Department of Virology, Paris Cité University and Cochin Hospital, AP-HP, Paris, France; 24 National Center for Genome Sequencing (CNSG), School of Medicine, University of Antioquia UdeA, Medellín, Colombia; 25 SISTEMIC Group, Department of Electronic Engineering, Faculty of Engineering, University of Antioquia UdeA, Medellín, Colombia; 26 Corporation for Biological Research (CIB), Medellín, Colombia; 27 University Hospital Erlangen, Institute of Virology, Friedrich-Alexander Universität Erlangen-Nürnberg, Erlangen, Germany; 28 Dermatology Section, Department of Internal Medicine, School of Medicine, University of Antioquia UdeA, Medellín, Colombia; 29 Dermatological Research Center (CIDERM), Medellín, Colombia; 30 Center for Molecular Medicine Cologne, University of Cologne, Cologne, Germany; 31 Neonatal Intensive Care Unit, San Matteo Research Hospital, Pavia, Italy; 32 School of Life Sciences, Swiss Federal Institute of Technology, Lausanne, Switzerland; 33 Host Pathogen Interactions program, Texas Biomedical Research Institute, San Antonio, TX USA; 34 Center for Cancer Research, Laboratory of Pathology, NCI, NIH, Bethesda, MD USA; 35 Department of Pathology, Ambroise Paré Hospital, AP-HP, Boulogne-Billancourt, France; 36 Laboratory of Clinical Immunology and Microbiology, Division of Intramural Research, NIAID, NIH, Bethesda, MD USA; 37 Department of Human Immunology, Sidra Medicine, Doha, Qatar; 38 College of Health and Life Sciences, Hamad Bin Khalifa University, Doha, Qatar; 39 Biomedical Research in Endstage and Obstructive Lung Disease Hannover (BREATH), Hannover, Germany; 40 Cluster of Excellence RESIST (EXC 2155), Hannover Medical School, Hannover, Germany; 41 Respiratory Diseases Department, FOCH Hospital, Suresnes, France; 42 Simone Veil Department of Health Sciences, Versailles Saint Quentin University, Montigny le Bretonneux, France; 43 Howard Hughes Medical Institute, New York, NY USA; 44 Department of Pediatrics, Necker Hospital for Sick Children, AP-HP, Paris, France

**Keywords:** Tuberculosis, Monocytes and macrophages, Tumour-necrosis factors

## Abstract

Severe defects in human IFNγ immunity predispose individuals to both Bacillus Calmette–Guérin disease and tuberculosis, whereas milder defects predispose only to tuberculosis^1^. Here we report two adults with recurrent pulmonary tuberculosis who are homozygous for a private loss-of-function *TNF* variant. Neither has any other clinical phenotype and both mount normal clinical and biological inflammatory responses. Their leukocytes, including monocytes and monocyte-derived macrophages (MDMs) do not produce TNF, even after stimulation with IFNγ. Blood leukocyte subset development is normal in these patients. However, an impairment in the respiratory burst was observed in granulocyte–macrophage colony-stimulating factor (GM-CSF)-matured MDMs and alveolar macrophage-like (AML) cells^2^ from both patients with TNF deficiency, TNF- or TNFR1-deficient induced pluripotent stem (iPS)-cell-derived GM-CSF-matured macrophages, and healthy control MDMs and AML cells differentiated with TNF blockers in vitro, and in lung macrophages treated with TNF blockers ex vivo. The stimulation of TNF-deficient iPS-cell-derived macrophages with TNF rescued the respiratory burst. These findings contrast with those for patients with inherited complete deficiency of the respiratory burst across all phagocytes, who are prone to multiple infections, including both Bacillus Calmette–Guérin disease and tuberculosis^3^. Human TNF is required for respiratory-burst-dependent immunity to *Mycobacterium tuberculosis* in macrophages but is surprisingly redundant otherwise, including for inflammation and immunity to weakly virulent mycobacteria and many other infectious agents.

## Abstract (web summary)

Human TNF is required for respiratory-burst-dependent immunity to *Mycobacterium tuberculosis* in macrophages but seems to be largely redundant physiologically.

## Main

Mendelian susceptibility to mycobacterial disease (MSMD) is characterized by clinical disease caused by weakly virulent mycobacteria, such as environmental mycobacteria or Bacillus Calmette–Guérin (BCG) vaccines, in otherwise healthy individuals^4^. Patients are also vulnerable to the more virulent *M. tuberculosis* and other intramacrophagic microorganisms^1^. MSMD is typically ‘isolated’ but can be ‘syndromic’ when associated with the frequent occurrence of at least one other phenotype, infectious or otherwise^5^. With one possible exception (ZNFX1 deficiency), all known genetic defects causing MSMD affect interferon-γ (IFNγ)-mediated immunity^6^. Variants of MSMD-causing genes disrupt the production of IFNγ (*IFNG*, *IL12B*, *IL12RB1*, *IL12RB2*, *IL23R*, *ISG15*, *MCTS1*, *RORC*, *TBX21*, *TYK2*), the response to IFNγ (*CYBB*, *JAK1*, *IFNGR1*, *IFNGR2*, *STAT1*, *USP18*) or both (*IRF1*, *IRF8*, *NEMO*, *SPPL2A*), or the recruitment of monocytes (*CCR2*)^7–14^. The penetrance and severity of mycobacterial disease are inversely correlated with levels of IFNγ activity, demonstrating that human IFNγ activity is a quantitative trait that governs antimycobacterial immunity^15^.

The phagocytic NADPH oxidase complex is a reactive oxygen species (ROS)-producing enzyme^16^. The production of ROS, including superoxide (O_2_^−^) and hydrogen peroxide (H_2_O_2_), is crucial for the phagocytic control of ingested bacteria, fungi and parasites^3^. Loss-of-function (LOF) variants in genes encoding the NADPH oxidase complex underlie chronic granulomatous disease (CGD), an inborn error of immunity (IEI) characterized by impaired or abolished ROS production in phagocytes^17^. CGD may be caused by variants affecting the core protein gp91^phox^ (encoded by *CYBB*) or one of its subunits (*CYBA*, *CYBC1*, *NCF1*, *NCF2* and *NCF4*). Patients with CGD are highly vulnerable to severe and/or recurrent infections with various pathogens, including mycobacteria such as BCG and *M. tuberculosis*^18,19^. Notably, patients with specific hypomorphic germline variants in *CYBB* selectively affecting ROS production in MDMs are highly susceptible to BCG disease or clinical tuberculosis (TB) but display no increase in the risk of other infections typically seen in patients with CGD^20^, suggesting that ROS production by macrophages is crucial for protective immunity to tuberculous mycobacteria.

It has been estimated that about 25% of the global population is infected with *M. tuberculosis*, but only 5–10% of infected individuals develop TB. The development of TB is strongly influenced by human genetic determinants as suggested by early genetic studies^21,22^. The frequency of BCG disease is about 1 in 50,000 individuals, implying that *M. tuberculosis* is at least 1,000 times more virulent than BCG. All patients with the MSMD phenotype with or without an MSMD genotype, and probably even all patients with an MSMD genotype with or without the MSMD phenotype, are therefore highly susceptible to *M. tuberculosis*^23^. Two rare monogenic IEIs have been reported to underlie TB in multiple kindreds: autosomal recessive (AR) complete IL-12Rβ1 and AR TYK2 deficiencies^24,25^. Homozygosity for the common *TYK2*^P1104A^ allele is the most common and penetrant aetiology of TB, underlying up to 1% of TB cases in European populations^4,26^. Finally, we recently described AR PD-1 deficiency and AR ITK deficiency as genetic aetiologies of TB^27,28^. Other IEIs, such as STAT1 gain of function (GOF)^29^, STAT3 GOF and PIK3CD GOF, also seem to confer a predisposition to TB, albeit through unknown mechanisms^5^. In this context, we studied two first-cousin Colombian adults with unexplained recurrent pulmonary TB but no adverse reaction to live BCG vaccination.

## Two patients with pulmonary TB

**Fig. 1:**
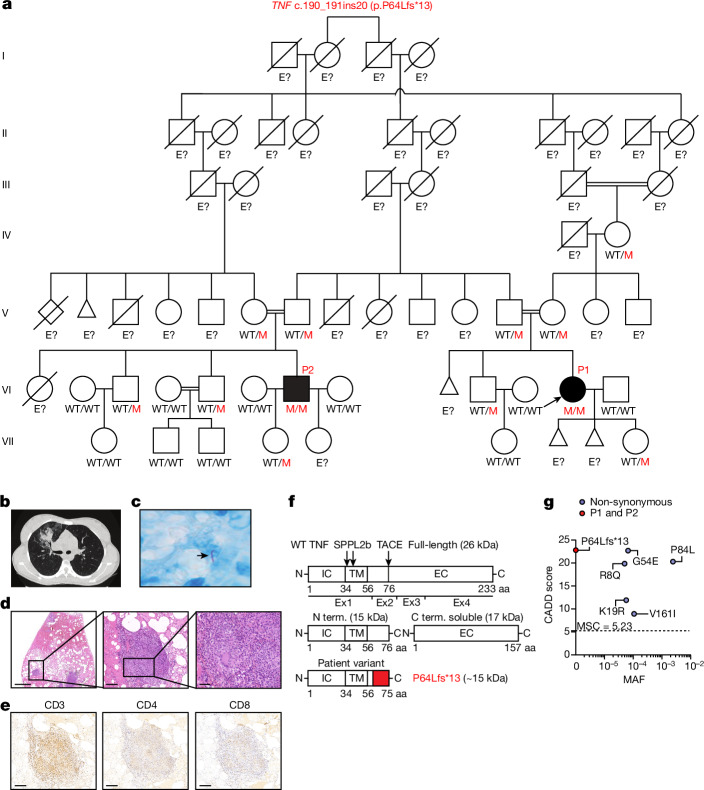
Identification of a biallelic *TNF* variant in two patients with pulmonary TB. **a**, Pedigree of two related kindreds showing familial segregation of the c.190_191ins20 (p.P64Lfs*13) *TNF* allele. Each generation is designated by a Roman numeral. Male and female individuals are represented by squares and circles, respectively. The filled boxes indicate individuals affected by TB and the crossed symbols indicate deceased individuals. ‘E?’ indicates an unknown *TNF* genotype. Red ‘M’ indicates the variant allele. The triangles indicate spontaneous abortion and the diamond indicates the death of an individual of unknown sex. **b**, Chest contrasted CT scan showing the pulmonary lesions of P1. **c**, Ziehl–Neelsen staining of a pulmonary biopsy specimen from P1 showing acid-fast bacilli (arrow). Data shown are representative of one independent experiment. **d**, Haematoxylin and eosin staining of a granuloma from P1 at different magnifications. Data shown are representative of one independent experiment. Scale bars, 2 mm, 200 µm and 50 µm (from left to right). **e**, Immunohistochemical staining for CD3, CD4 and CD8, indicating the presence of T cells within the granuloma of P1. Data shown are representative of one independent experiment. Scale bars, 200 µm. **f**, Schematic of the full-length and cleaved TNF proteins, with the intracellular (IC), transmembrane (TM) and extracellular (EC) domains indicated. The red part of the mutant TNF protein (P64Lfs*13) corresponds to the amino acids inserted due to the frameshift variant. aa, amino acids. N term. and C term., N-terminal and C-terminal fragments. **g**, The minor allele frequency (MAF; gnomAD, v.2.1.1) and combined annotation-dependent depletion (CADD; v.1.6) score for biallelic non-synonymous TNF variants reported in gnomAD v.2.1.1 or found in P1 and P2. The MSC is indicated by a dotted line.

We studied two related patients from a large consanguineous family from Colombia (Fig. 1a; see the ‘Extended case reports for P1 and P2’ section of the Methods). P1 is a 28-year-old woman. She was diagnosed with pulmonary TB at the age of 19 years, presenting with dry cough, pleuritic pain, fever (40 °C), night sweats, dyspnoea and unexplained weight loss. Chest X rays and computed tomography (CT) revealed multiple pulmonary micronodules and nodular lesions in the right lung (Fig. 1b). A segmental lobectomy of the right upper lobe was performed (Fig. 1c–e). Histological analysis showed well-constituted, paucibacillary granulomas, some of which were necrotic (Fig. 1d). Immunostaining for CD3, CD4 and CD8 revealed a retention of T cells at the granuloma site and Ziehl–Neelsen staining detected acid-fast bacteria (Fig. 1c,e). Cultures of sputum, bronchoalveolar lavage fluid and pulmonary nodule samples were positive for *M. tuberculosis*. The patient was treated for 12 months with isoniazid (H), rifampicin (R), pyrazinamide (Z) and ethambutol (E) (HRZE), leading to complete remission. Then, 14 months later, P1 was again diagnosed with pulmonary TB. HRZE treatment was administered for 30 days, resulting in complete remission. At the age of 22 years, at 28 weeks of pregnancy, P1 was hospitalized for septic shock, presenting with leukocytosis (25.7 × 10^9^ cells per litre) with neutrophilia (18.80 × 10^9^ cells per litre), high C-reactive protein concentration (CRP; 8.7 mg dl^−1^) and persistent fever (>38 °C). Clinical chorioamnionitis necessitated an emergency caesarean section. Blood cultures were positive for *Listeria monocytogenes*. The patient was treated with trimethoprim-sulfamethoxazole and ampicillin for 7 days, with a favourable outcome.

P2 is a 36-year-old man and first cousin of P1. At the age of 18 years, he presented with left pulmonary TB with pleural effusion, which was treated for 6 months with HRZE. Then, 8 months later, he was hospitalized due to a relapse of TB.

**Extended Data Fig. 1:**
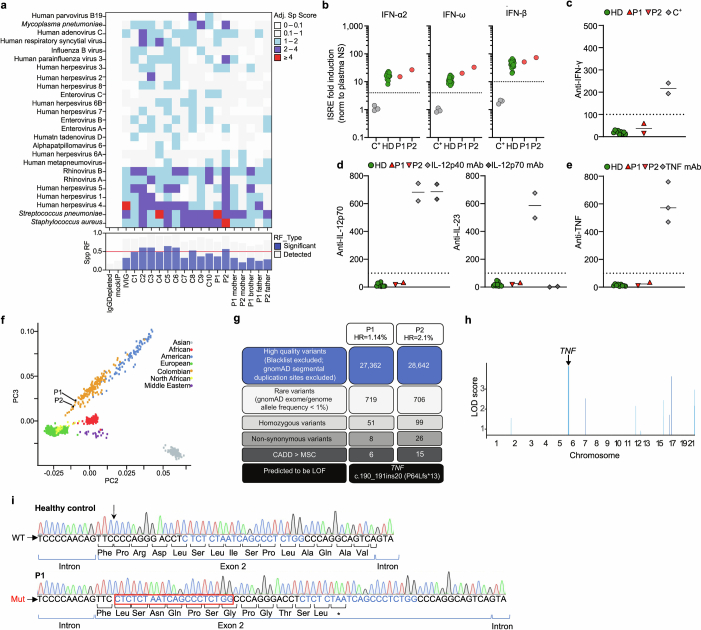
Immunological and genetic features of human TNF deficiency. **a**, Heatmap indicating scores equivalent to the count of peptides displaying significant enrichment for a given species in serum samples from healthy controls, P1, P2 and their relatives. IgG-depleted serum (IgGDepleted) and pooled plasma used for intravenous immunoglobulin (IVIG) therapy were used as negative and positive controls, respectively. **b**, Luciferase-based neutralization assay for the detection of auto-Abs neutralizing IFN-α2, IFN-ω and IFN-ß in plasma from healthy controls (HD; *n* = 18), P1 and P2, and individuals known to have neutralizing auto-Abs (C^+^; *n* = 3). The dashed line indicates the threshold for neutralizing activity. **c**, Detection of auto-Abs against IFN-γ in plasma from healthy controls (*n* = 9), P1, P2 and an individual known to have anti-IFN-γ auto-Abs (C^+^). **d**, Detection of auto-Abs against IL-12p70 and IL-23 in plasma from healthy controls (*n* = 9), P1and P2. Monoclonal antibodies (mAb) against IL-12p40 and IL-12p70 were used as positive controls. **e**, Detection of auto-Abs against TNF in plasma from healthy controls (*n* = 9), P1 and P2. **f**, Principal component (PC) analysis on WES data from P1, P2, our in-house database, and samples from the 1000 Genomes Project. **g**, WES analysis of P1 and P2, with homozygosity rates (HR) indicated. LOF, loss-of-function **h**, Linkage analysis. An arrow indicates the chromosome on which the *TNF* gene is located. **i**, Sanger sequencing of the region containing the *TNF* variant for a wild-type healthy control and P1.

No other unusual infections were reported in P1 or P2. The QuantiFERON-TB Gold test and the Mantoux tuberculin skin test (TST) were positive for both patients, but negative for their parents and the brother of P1. Both patients were vaccinated with BCG (Pasteur strain) without adverse effects. None of their relatives had any history of severe infectious disease. VirScan analysis of P1 and P2 confirmed normal antibody repertoires against common pathogens, demonstrating intact T cell and B cell functions for seroconversion after vaccination and immunity to common infections (Extended Data Fig. 1a and Extended Data Table 1). Blood samples from the patients contained no autoantibodies neutralizing type I or type II interferons, IL-12p70, IL-23 or TNF itself (Extended Data Fig. 1b–e).

## Homozygosity for a *TNF* frameshift variant

We performed whole-exome sequencing (WES) and genome-wide linkage analysis of P1, P2, their parents and P1’s unaffected brother. Principal component analysis of the WES data confirmed the Colombian ancestry of these individuals (Extended Data Fig. 1f). The high homozygosity rates of P1 (1.14%) and P2 (2.1%) confirmed parental consanguinity. Assuming genetic homogeneity, we searched for AR genetic aetiologies (Extended Data Fig. 1g). By filtering for non-synonymous variants with a minor allele frequency (MAF) of <0.01 in gnomAD v.2.1.1 and a combined annotation dependent-depletion (CADD) score above the mutation significance cut-off (MSC), we identified a 20-nucleotide insertion (c.190_191ins20) in exon 2 of *TNF* common to the two patients (Extended Data Tables 2 and 3). This insertion results in a frameshift, generating a 12-amino-acid extension and a premature stop codon 13 amino acids downstream (p.P64Lfs*13) (Fig. 1f). Genome-wide linkage analysis yielded a maximum logarithm of the odds score for a region encompassing *TNF* on chromosome 6 (Extended Data Fig. 1h). Sanger sequencing confirmed that the patients were homozygous for the variant, whereas their unaffected relatives were heterozygous (Fig. 1a and Extended Data Fig. 1i). TNF is a potent pro-inflammatory cytokine produced principally by macrophages as a transmembrane precursor organized into stable homotrimers. This membrane-integrated form is then cleaved by a metalloprotease, leaving an N-terminal fragment and a soluble, secreted C-terminal fragment^30^ (Fig. 1f). The variant present in the patients is predicted to affect both TNF fragments, as the resulting frameshift and premature stop codon lead to a loss of the TACE-cleavage site. The p.P64Lfs*13 variant is private and is predicted to be deleterious, with a CADD score of 22.5, well above the MSC for *TNF* (Fig. 1g). Five other variants are present in the homozygous state in the BRAVO/TOPmed freeze 8, ATAV, ExAC and gnomAD v.2.1.1 databases and there are only two predicted LOF (pLOF) variants of this gene in gnomAD v.2.1.1, with a cumulative MAF of 8.27 × 10^−6^ (Fig. 1g). No individuals homozygous for pLOF variants were found in gnomAD or any other public WES database. *TNF* has a gene damage index of 0.55, a residual variation intolerance score of −0.189 and a probability of LOF intolerance of 0.8, all suggestive of a high degree of intolerance to protein-truncating variants. Consistently, the consensus negative selection score of *TNF* is low (−0.98)^31^. Together, these findings suggest that the private c.190_191ins20 *TNF* variant is deleterious and that it may underlie recurrent TB in these two patients.

## The patients’ *TNF* variant is LOF

**Fig. 2:**
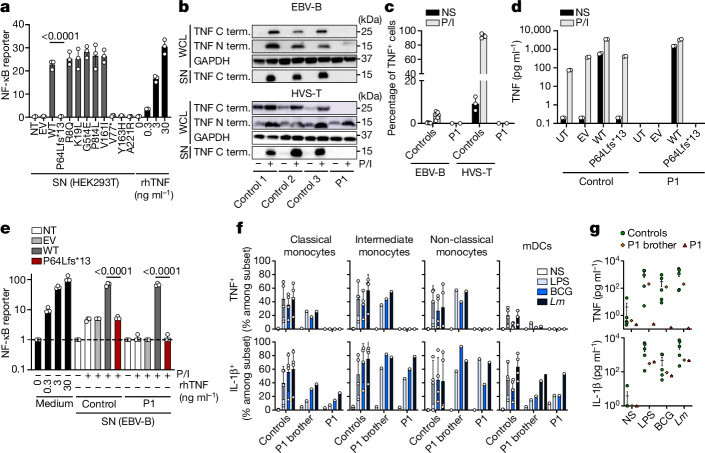
The patients’ variant results in the loss of TNF production. **a**, NF-κB-luciferase reporter activity in HEK293T cells stimulated with recombinant human TNF (rhTNF) or supernatants from HEK293T cells transfected with the patients’ TNF variant or variants present in the homozygous state in gnomAD v.2.1. Data are mean ± s.d. from three independent experiments. EV, empty vector; NT, not transfected; SN, supernatant. **b**, Western blot analysis of supernatants and whole-cell lysates (WCL) from EBV-B and HVS-T cells from healthy controls and P1 with and without PMA and ionomycin (P/I) stimulation. Representative image from three independent experiments. **c**, Detection of intracellular TNF by flow cytometry in EBV-B cells (*n* = 7) and HVS-T cells (*n* = 3) from healthy controls and P1 without (NS) or with PMA and ionomycin stimulation. Data are mean ± s.d. from two independent experiments. **d**, TNF production by EBV-B cells from a healthy control or P1 left untransduced (UT), or transduced with an EV or with plasmids encoding WT or variant TNF, with or without PMA and ionomycin stimulation. Data are mean ± s.d. from two independent experiments. **e**, NF-κB-luciferase reporter activity in HEK293T cells stimulated for 24 h with recombinant human TNF or supernatants of transduced EBV-B cells as in **d**. Data are mean ± s.d. from three independent experiments. **f**, The frequency of cells producing TNF and IL-1β in response to LPS, BCG or *L. monocytogenes* (*Lm*) for the indicated subsets from P1, her brother and healthy controls (*n* = 4). Data of healthy controls (*n* = 4) are mean ± s.d. mDCs, myeloid dendritic cells. **g**, Secretion of TNF and IL-1β by total PBMCs from P1, her brother and healthy controls (*n* = 5) after stimulation with LPS, BCG or *L. monocytogenes.* Significance was assessed using two-tailed Mann–Whitney *U* tests, comparing between the WT and the patients’ variant (**a** and **e**).

**Extended Data Fig. 2:**
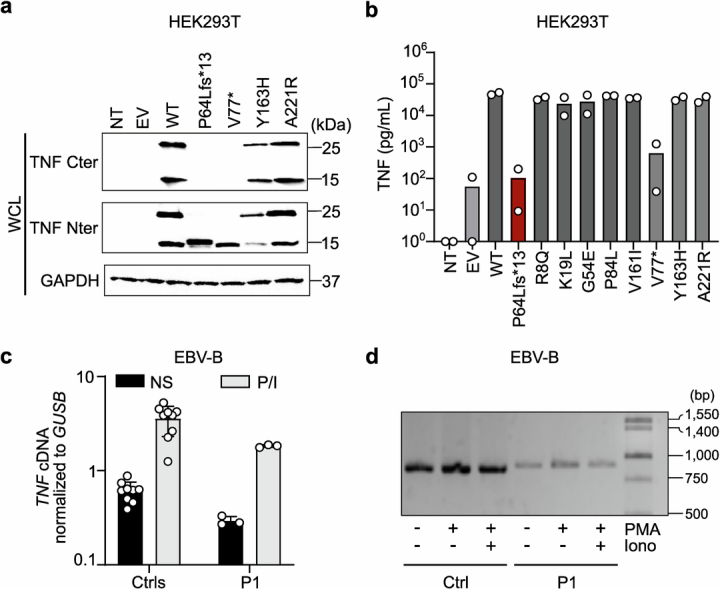
The patients’ variant results in conserved mRNA production and a loss of soluble TNF. **a**, Western blot of whole-cell lysates (WCL) from HEK293T cells transfected with plasmids encoding wild-type or variant TNF. NT, not transfected; EV, empty vector. The results shown are representative of three independent experiments. **b**, TNF detection by bead-based immunoassay on supernatants from HEK293T cells transfected as in **(a)**. Data are mean ± s.d. from two independent experiments. **c**, *TNF* expression assessed by RT-qPCR on cDNA from EBV-B cells from P1 and healthy controls (*n* = 3) left non-stimulated (NS) or stimulated with PMA-ionomycin (P/I). Data are mean ± s.d. from three independent experiments. **d**, Agarose gel showing *TNF* amplification from cDNA from the EBV-B cells of a healthy control or P1 with or without stimulation. Data shown are representative of three independent experiments.

We investigated the effect of *TNF* variants on protein levels by transfecting human embryonic kidney 293T (HEK293T) cells with wild-type (WT) or variant *TNF*. The previously described p.Y163H and p.A221R variants^32^, which encode proteins that do not bind to TNFR1 and TNFR2, and the p.V77* variant, were used as negative controls. Western blot analysis of whole-cell lysates resulted in the detection of the full-length (25 kDa) TNF and a cleaved N-terminal fragment (15 kDa) for the WT, whereas only a 15 kDa protein was detected for the p.P64Lfs*13 variant (Extended Data Fig. 2a). An antibody directed against the C terminus detected two bands for the WT, corresponding to the full-length TNF and the cleaved C-terminal fragment, whereas no bands were detected for the p.P64Lfs*13 variant, ruling out reinitiation of translation. No increase in TNF levels was observed in the supernatant of HEK293T cells transfected with the patients’ variant (Extended Data Fig. 2b). We evaluated the function of the proteins encoded by the various TNF variants, by assessing the capacity of supernatants from transfected HEK293T cells to induce NF-κB-dependent transcription in a luciferase reporter assay (Fig. 2a). No induction of luciferase activity was detected after treatment with the supernatants of cells transfected with the patients’ variant, whereas strong luciferase activity was observed after treatment with the supernatants of cells transfected with the WT or public variants. Thus, in contrast to the five variants found in the homozygous state in public databases, the patients’ variant abolishes the secretion of mature TNF and is LOF.

## The *TNF* genotype underlies complete TNF deficiency

**Extended Data Fig. 3:**
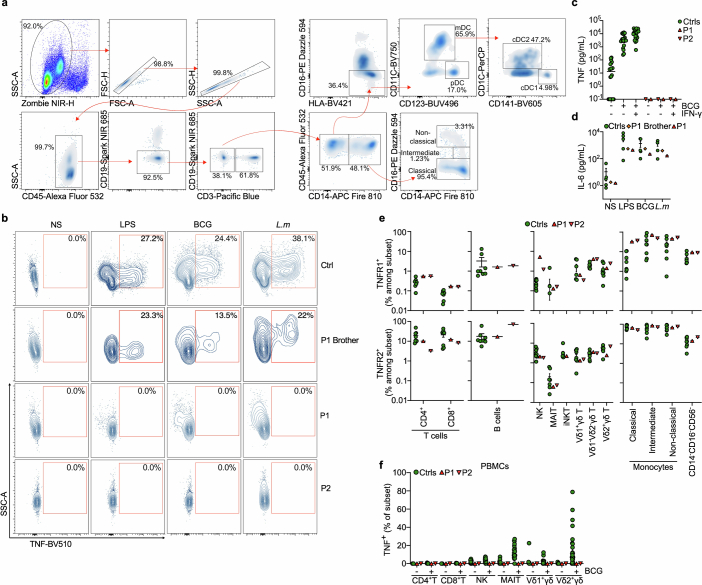
TNF production by the patients’ primary cells. **a**, Gating strategy related to Fig. 2e for identification of the dendritic cell (DC) and monocyte subsets. **b**, TNF production by classical monocytes from a local control (Ctrl), P1 and P2 and the healthy brother of P1 without (NS) and with stimulation with LPS, BCG or *L. monocytogenes* (*L.m*). **c**, TNF secretion into whole blood after stimulation with BCG or IFN-γ for healthy controls (*n* = 22), P1 and P2. **d**, IL-6 secretion by total PBMCs from P1, her brother and healthy controls (*n* = 5) after stimulation with LPS, BCG or *L.m*. Error bars indicate the mean ± s.d. **e**, Frequency of TNFR1^+^ and TNFR2^+^ cells in the indicated subsets of PBMCs from healthy controls (*n* = 8), and TNF-deficient patients. Error bars indicate the mean ± s.d. **f**, Frequency of TNF^+^ cells in the indicated subsets of PBMCs with and without stimulation with BCG, for healthy controls (*n* = 22), P1 and P2. mDC, myeloid dendritic cells; MAIT, mucosal-associated invariant T cells; iNKT, invariant natural killer T cells.

We evaluated the effect of the patients’ biallelic *TNF* variant on mRNA and protein levels in Epstein–Barr-virus-transformed B (EBV-B) and *Herpesvirus saimiri*-transformed T (HVS-T) cell lines from P1. Quantitative PCR with reverse transcription (RT–qPCR) analysis of the EBV-B cells of P1 showed normal *TNF* mRNA expression (Extended Data Fig. 3c,d). Western blot analysis, using antibodies directed against the N terminus of TNF, of whole-cell lysates and supernatants from the EBV-B and HVS-T cells of P1 and healthy controls revealed the presence of a truncated protein in the cells of P1, whereas no protein was detected when using antibodies directed against the C terminus of TNF (Fig. 2b). Intracellular flow cytometry staining for TNF on phorbol 12-myristate 13-acetate (PMA)/ionomycin-stimulated HVS-T and EBV-B cells confirmed the absence of TNF expression in the cells of P1 (Fig. 2c). Enzyme-linked immunosorbent assay (ELISA) analysis of supernatants from control and patient EBV-B cells also showed that P1’s cells did not secrete TNF (Fig. 2d). Lentiviral transduction of P1’s EBV-B cells with WT *TNF* rescued TNF production and function (Fig. 2d,e). These data confirm that the patients’ variant impairs TNF production and abolishes TNF secretion and that the patients therefore have AR complete TNF deficiency. The occurrence of TB in patients on TNF blockade is well documented, suggesting that these two patients had TB because of their TNF deficiency^33^. Together, these findings suggest that the TNF genotype is causal for the TB phenotype after exposure to *M. tuberculosis*.

## The patients’ leukocytes lack TNF

TNF is produced principally by mononuclear myeloid cells in response to microbial stimuli (Fig. 2f). We measured the TNF production of primary myeloid cells from P1 in response to stimulation with lipopolysaccharide (LPS), BCG or *L. monocytogenes*. Intracellular TNF levels increased in monocytes and myeloid dendritic cells (DCs) of healthy controls after stimulation (Fig. 2f and Extended Data Fig. 3a,b). By contrast, no TNF production was observed in any of the myeloid subsets from P1 or in classical monocytes from P2 (Fig. 2f and Extended Data Fig. 3b). Consistently, TNF was undetectable in the supernatants of stimulated peripheral blood mononuclear cells (PBMCs) from P1 or IFNγ + BCG-stimulated whole blood from both patients (Fig. 2g and Extended Data Fig. 3c). The production and secretion of other pro-inflammatory cytokines, such as IL-1β and IL-6, were normal in the myeloid cells and total PBMCs of P1 (Fig. 2g and Extended Data Fig. 3d). For the healthy controls, TNF production was highest in monocytes, with various lymphocyte subsets producing only small amounts of TNF after exposure to BCG. Lymphocytes from both patients displayed a complete abolition of TNF production but conserved expression of the TNF receptors 1 and 2 (TNFR1/2; Extended Data Fig. 3e,f). Both the myeloid and lymphoid subsets of both patients displayed complete TNF deficiency but conserved TNFR1 and TNFR2 expression.

## Normal leukocyte development

**Extended Data Fig. 4:**
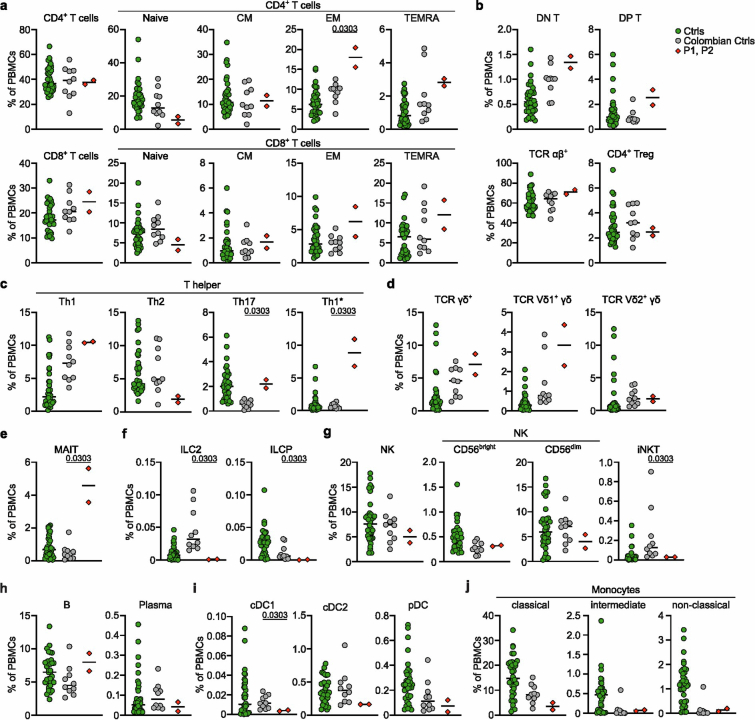
Deep immunophenotyping by spectral flow cytometry for TNF-deficient patients. Frequency of **(a)** CD4^+^ T cells and CD8^+^ T cells and their subsets, **(b)** double-negative (DN), double-positive (DP), T-cell receptor (TCR) αβ^+^ and regulatory T cells (Treg), **(c)** T helper (Th) cells and their subsets, **(d)** TCR γδ^+^ T cells, **(e)** mucosal-associated invariant T cells (MAIT), **(f)** innate lymphoid cells (ILC), **(g)** NK cells and their subsets, **(h)** B cells and plasma cells, **(i)** dendritic cell (DC) subsets, **(j)** and monocytes and their subsets in healthy adult controls (*n* = 36), healthy, age-matched controls from Colombia (*n* = 10), P1 and P2, as determined by spectral flow cytometry. CM, central memory; EM, effector memory; TEMRA, effector memory re-expressing CD45RA, ILCP, innate lymphoid cell precursor, iNKT, invariant natural killer T cells; mDC, myeloid dendritic cells; cDC, conventional dendritic cells; pDC, plasmacytoid dendritic cells. Significance was assessed using two-tailed Mann-Whitney *U* tests (**a, c**, **e**, **f**, **g** and **i**).

Some patients on TNF blockade develop neutropenia during treatment^34^. We therefore analysed the effect of TNF deficiency on leukocyte development. Complete blood-cell counts at various ages were normal for P1 and P2 (Extended Data Table 4). We used spectral flow cytometry to investigate the various PBMC subsets of P1 and P2 after complete remission of TB had been achieved (Extended Data Fig. 4). Both patients had similar proportions of total CD4^+^ and CD8^+^ T cells, and similar proportions of the naive, central memory and effector memory re-expressing CD45RA (TEMRA) subsets to healthy controls matched for ethnicity and age (Extended Data Fig. 4a). Their proportions of effector memory CD4^+^ cells were high, whereas the proportions of naive CD8^+^ cells, regulatory T cells, and double-negative and double-positive T cells were within the normal range (Extended Data Fig. 4a,b). The proportions of T helper cell subsets were normal (T_H_1, T_H_2) or high (T_H_17, T_H_1*) in both patients (Extended Data Fig. 4c). The frequencies of γδ T cells, Vδ1^+^ γδ T cells and Vδ2^+^ γδ T cells were unaffected by TNF deficiency, whereas the frequencies of mucosal-associated invariant T cells were high (Extended Data Fig. 4d,e). The frequencies of type 2 innate lymphoid cells and innate lymphoid cell precursors were low, as was the proportion of invariant natural killer T (iNKT) cells (Extended Data Fig. 4f–h). The proportions of B cells and NK cells and their subsets were normal in the patients (Extended Data Fig. 4f–h). The frequencies of conventional type 1 DCs (cDC1 cells) were low but the proportions of cDC2 cells and plasmacytoid DCs were unaffected, as were the frequencies of monocytes and their subsets (Extended Data Fig. 4i,j). Overall, these data suggest that complete TNF deficiency does not alter the development of the major myeloid and lymphoid subsets in the blood.

## Normal IFNγ immunity in the patients

**Extended Data Fig. 5:**
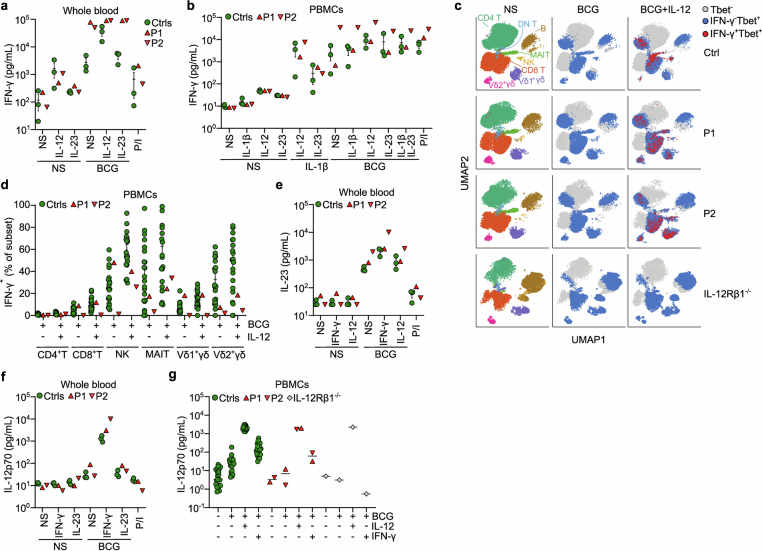
Production of and response to IFN-γ in PBMCs from TNF-deficient patients. **a**, IFN-γ secretion into whole blood for healthy controls (*n* = 3), P1 and P2, with and without (NS) stimulation with IL-12, IL-23, BCG or PMA/ionomycin (P/I). Error bars indicate the mean ± s.d. **b**, IFN-γ secretion by PBMCs from healthy controls (*n* = 3), P1 and P2, after stimulation with IL-1β, IL-12, IL-23, BCG or P/I. Error bars indicate the mean ± s.d. **c**, UMAP clustering of PBMCs from two healthy controls, P1, P2 and an IL-12Rβ1-deficient patient profiled by flow cytometry after stimulation with BCG or BCG + IL-12. Cell populations are annotated, as are cells expressing T-bet and IFN-γ. **d**, Frequency of IFN-γ^+^ cells in the indicated subsets of PBMCs with and without stimulation with BCG or BCG + IL-12, for healthy controls (*n* = 22), P1 and P2. Error bars indicate the mean ± s.d. **e**, IL-23 secretion into whole blood for healthy controls (*n* = 3), P1 and P2 after stimulation with IFN-γ, IL-12 and BCG, or P/I. Error bars indicate the mean ± s.d. **f**, IL-12p70 secretion into whole blood after stimulation with IFN-γ, IL-23 and BCG, or P/I, for healthy controls (*n* = 3), P1 and P2. **g**, IL-12p70 production by PBMCs from healthy controls (*n* = 22), P1, P2 and an IL-12Rβ1-deficient patient, after stimulation with BCG, IFN-γ and IL-12.

The known genetic aetiologies of MSMD and TB impair the production of or response to IFNγ^5^. TNF is produced predominantly by myeloid cells and its production is increased by IFNγ^35^. We therefore investigated the effect of TNF deficiency on IFNγ immunity. Whole-blood IFNγ levels after stimulation with IL-12, IL-23 and BCG were similar to or higher than those of healthy controls (Extended Data Fig. 5a). Total PBMCs stimulated with IL-1β, IL-12, IL-23 or BCG secreted large amounts of IFNγ, similar to those observed for healthy controls (Extended Data Fig. 5b). P1 and P2 had normal proportions of IFNγ^+^, IFNγ^+^T-bet^+^CD4^+^ and CD8^+^ T cells, NK cells, iNKT cells, mucosal-associated invariant T cells, and Vγδ1^+^ and Vγδ2^+^ T cells after stimulation with BCG and IL-12 (Extended Data Fig. 5c,d). Thus, TNF deficiency does not affect IFNγ production by lymphocytes in vitro. We next evaluated the response to IFNγ by measuring the levels of IL-23 and IL-12p70 in whole blood from P1 and P2 after stimulation with IFNγ and BCG (Extended Data Fig. 5e,f). The patients’ cells secreted normal amounts of IL-23 and IL-12p70 in response to all of the stimuli tested. Moreover, the production of IL-12p70 by PMBCs from P1 and P2 was normal after stimulation with BCG and BCG–IFNγ, confirming the normal global response to IFNγ in vitro (Extended Data Fig. 5g). The production of, and response to, IFNγ were therefore normal in blood leukocytes from both of the patients with TNF deficiency.

## scRNA-seq of TNF-deficient leukocytes

**Extended Data Fig. 6:**
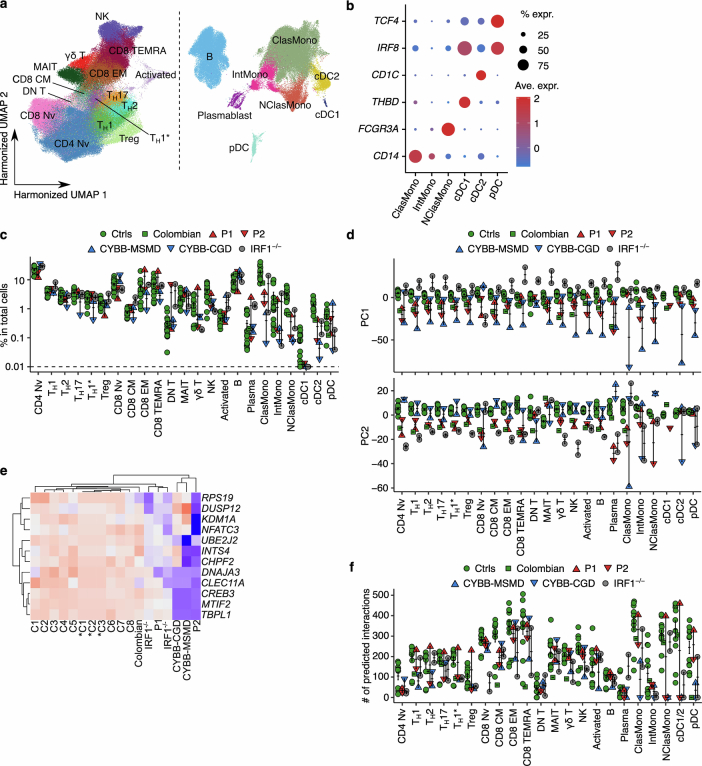
Single-cell transcriptomic analysis of TNF-deficient leukocytes. Cryopreserved PBMCs from P1 and P2 were analysed with cells from healthy adult controls (*n* = 11), one healthy Colombian control, two patients with *CYBB* variants (underlying MSMD or CGD) and two patients with IRF1 deficiency. **a**, Clustering analysis. After batch correction with Harmony, clusters were identified manually with the aid of the SingleR pipeline informed by the MonacoImmuneDataset. **b**, Expression levels of representative marker genes for myeloid lineage subsets. **c**, Cell abundance from healthy adult controls (*n* = 11), one Colombian control, TNF-deficient (*n* = 2), IRF1-deficient (*n* = 2) and CYBB-deficient (*n* = 2) patients. **d**, Pseudobulk principal component analysis from healthy adult controls (*n* = 11), one Colombian control, TNF-deficient (*n* = 2), IRF1-deficient (*n* = 2) and CYBB-deficient (*n* = 2) patients. **e**, Heat map showing the levels of expression of genes from the NPM1-target geneset in non-classical monocytes, as determined by scRNA-sequencing. Three healthy adult controls (*C1-*C3) were used for batch correction. **f**, Intercellular communication analysis with CellChat from healthy adult controls (*n* = 11), one Colombian control, TNF-deficient (*n* = 2), IRF1-deficient (*n* = 2) and CYBB-deficient (*n* = 2) patients. In (**c, d** and **f**), bars represent the mean and s.e.m. Data shown are representative of one independent experiment.

We determined the molecular basis of TB in patients with inherited TNF deficiency by studying PBMCs from P1 and P2 using single-cell RNA sequencing (scRNA-seq). We also studied PBMCs from one healthy Colombian control individual, two patients with specific variants in *CYBB* causing MSMD or CGD, and two previously published patients with IRF1 deficiency^7^. Unsupervised clustering analysis identified 17 lymphoid and 6 myeloid leukocyte subsets (Extended Data Fig. 6a,b). Both patients with TNF deficiency had slightly lower counts of classical monocytes compared with the healthy adult controls (Extended Data Fig. 6c). P2 also had low counts of non-classical monocytes and cDC2 cells (Extended Data Fig. 6c). Pseudobulk principal component analysis for each leukocyte subset showed normal PC1 but different PC2 for plasmablasts, non-classical monocytes and cDC1 cells from the patients with TNF deficiency, as for the two IRF1-deficient patients (Extended Data Fig. 6d). Gene set enrichment analysis (GSEA) identified the NPM1-target gene set as the only gene set that was significantly downregulated in non-classical monocytes from P1 and P2 but not in those from the Colombian control individual, relative to the healthy adult controls. However, the same gene set was downregulated in non-classical monocytes from the two patients with IRF1 deficiency and from patients with *CYBB* variants underlying CGD or MSMD, suggesting that this phenotype is not specific to TNF deficiency (Extended Data Fig. 6e). The total number of inferred intercellular interactions, estimated by CellChat, remained within the normal range for all leukocyte subsets analysed (Extended Data Fig. 6f). Overall, inherited TNF deficiency led to only a subtle disturbance of transcriptional profiles in non-classical monocytes at steady state in vivo, whereas other lymphoid and myeloid leukocyte subsets were spared.

## Low ROS levels in TNF-deficient macrophages

**Fig. 3:**
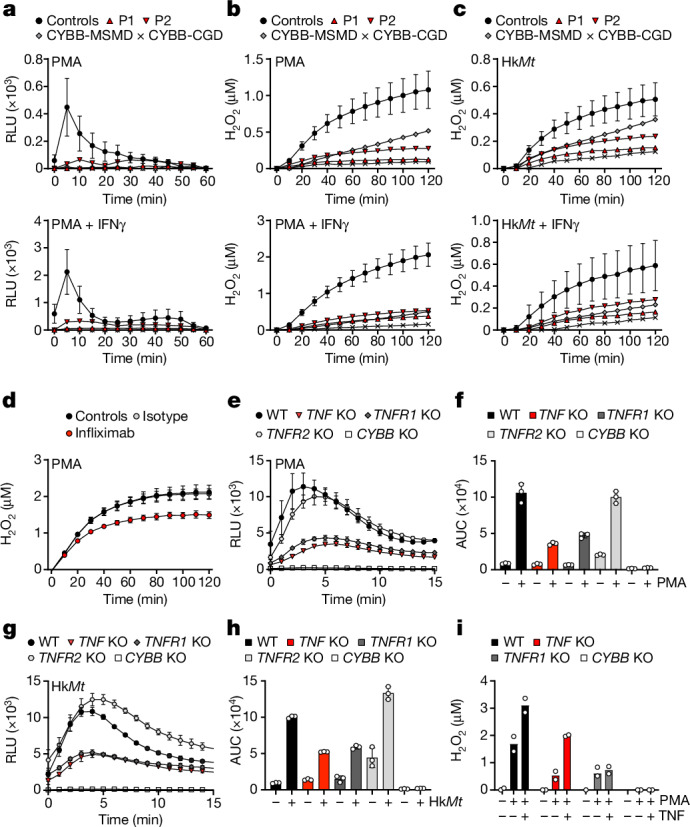
Reduced NADPH oxidase activity in TNF-deficient GM-CSF-matured macrophages. **a**, The production of O_2_^−^, in relative luminescence units (RLU), by MDMs matured in the presence of GM-CSF for healthy controls (*n* = 5), patients with TNF deficiency (P1, P2) and patients with variants in *CYBB* underlying MSMD (*n* = 1) or CGD (*n* = 1), after stimulation with PMA with or without IFNγ. Data of healthy controls (*n* = 5) are mean ± s.d. **b**,**c**, The extracellular production of H_2_O_2_ in GM-CSF-matured MDMs for healthy controls (*n* = 5), patients with TNF deficiency (P1 and P2) and patients with variants in *CYBB* underlying MSMD (*n* = 1) or CGD (*n* = 1), after stimulation with PMA (**b**) or in response to serum-opsonized heat-killed *M. tuberculosis* (hk*Mt*) (**c**) with or without IFNγ. Data of healthy controls (*n* = 5) are mean ± s.d. **d**, H_2_O_2_ release by GM-CSF-matured MDMs from healthy controls differentiated in culture medium in the presence of infliximab or the corresponding isotype control. One representative experiment of two independent experiments with three biological replicates. Data are mean ± s.d. **e**, O_2_^−^ production in response to PMA stimulation by iPS-cell-derived macrophages and iPS-cell-derived macrophages gene-edited to KO *TNF*, *TNFR1*, *TNFR2* or *CYBB* expression. One representative experiment of three independent experiments with three biological replicates. Data are mean ± s.d. **f**, Area-under-the-curve (AUC) analysis of O_2_^−^ production by iPS-cell-derived macrophages as in **e** with or without PMA stimulation. Data are mean ± s.d. of three independent experiments. **g**, O_2_^−^ production by iPS-cell-derived macrophages in response to stimulation with heat-killed *M. tuberculosis*. One representative experiment of three independent experiments with three biological replicates. Data are mean ± s.d. **h**, AUC analysis of O_2_^−^ production by iPS-cell-derived macrophages as in **g** with or without stimulation with heat-killed *M. tuberculosis*. Data are mean ± s.d. of three independent experiments. **i**, Extracellular H_2_O_2_ release by iPS-cell-derived macrophages in response to PMA with or without TNF. *n* = 2 independent experiments.

**Extended Data Fig. 7:**
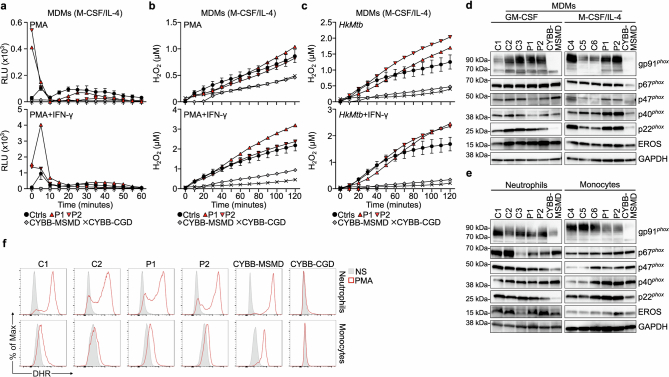
NADPH oxidase expression and activity in TNF-deficient phagocytes. **a**, Production of O_2_^−^, in relative luminescence units (RLU), by MDMs matured in the presence of M-CSF and IL-4, for healthy controls (*n* = 5), TNF-deficient patients, and patients with variants in *CYBB* underlying MSMD (*n* = 1) or CGD (*n* = 1) after stimulation with PMA with or without IFN-γ. For healthy controls (*n* = 5) the data are mean ± s.d. **b**, Extracellular production of H_2_O_2_ in M-CSF/IL-4-matured MDMs as in **(a)** after stimulation with PMA with or without IFN-γ. Data are mean ± s.d. **c**, H_2_O_2_ release by M-CSF/IL-4-matured MDMs as in **(a)** in response to serum-opsonized heat-killed *Mtb* (*HkMtb*) stimulation with or without IFN-γ. Data are mean ± s.d. **d**, Western blot of whole-cell lysates of MDMs matured by incubation with GM-CSF or M-CSF/IL-4, for healthy controls, P1, P2 and a patient with a *CYBB* variant underlying MSMD (CYBB-MSMD), for NADPH oxidase subunits. Data shown are representative of one independent experiment. **e**, Western blot for NADPH oxidase subunits on whole-cell lysates of neutrophils and monocytes from patients and controls. Data shown are representative of one independent experiment. **f**, Intracellular ROS production by neutrophils and monocytes, measured by DHR, after stimulation with PMA in two healthy controls, TNF-deficient patients and patients with variants in *CYBB* underlying MSMD (*n* = 1) or CGD (*n* = 1). Representative data from two independent experiments are shown.

**Extended Data Fig. 8:**
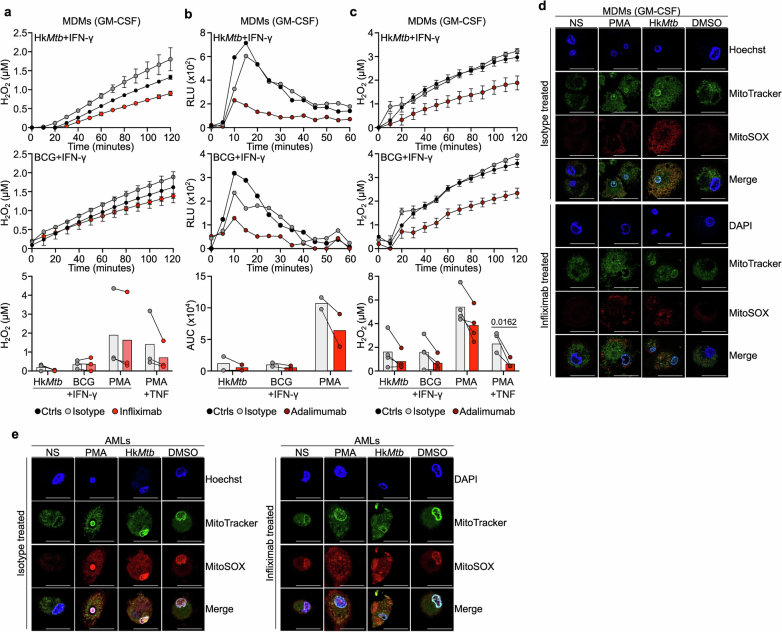
Loss of TNF signalling in AML cells impairs NADPH oxidase activity in response to physiological stimuli. **a**, Extracellular production of H_2_O_2_ by GM-CSF-matured MDMs from healthy controls treated with infliximab or isotype control after stimulation with (top) heat-killed *Mtb* (Hk*Mtb*) or (middle) live BCG. Data are mean ± s.d. and representative of three independent experiments. Bottom: H_2_O_2_ release by GM-CSF-matured MDMs (*n* = 3) after 30 min of stimulation. **b**, Production of O_2_^−^, in relative luminescence units (RLU), by GM-CSF-matured MDMs from healthy controls treated with adalimumab or isotype control, stimulated as in **(a)**. Representative data from *n* = 3 independent experiments are shown. Bottom: AUC analysis of O_2_^−^ production by human GM-CSF-matured MDMs (*n* = 3) in response to Hk*Mtb*, live BCG or PMA, with IFN-γ stimulation for 24 h. **c**, Production of H_2_O_2_ by GM-CSF-matured MDMs from healthy controls treated as in **(b)** and stimulated as in **(a)**. Data are mean ± s.d. and representative of three independent experiments. **(d-e)** Mitochondrial ROS levels were assessed with MitoSOX indicators in healthy control (**d**) GM-CSF-matured MDMs or **(e)** AML cells treated with infliximab or isotype control. The images shown are representative of three independent experiments. NS, not stimulated. Scale bar 20 µm. Significance was assessed by two-tailed paired **(c)** *t*-test; *p < 0.05.

The production of ROS by human macrophages is essential for host defence against both BCG and *M. tuberculosis*^20^. TNF signalling through TNFR1 has been reported to be important for NADPH oxidase complex activation, as it enhances the incorporation of FAD into the NADPH oxidase enzymes^36^. We therefore assessed ROS production in MDMs from P1 and P2. O_2_^−^ production and extracellular H_2_O_2_ release in response to stimulation with PMA and IFNγ were highly impaired in GM-CSF-matured MDMs from P1 and P2 (Fig. 3a,b). The cells of P1 and P2 displayed similar impairments of extracellular H_2_O_2_ production with serum-opsonized heat-killed *M. tuberculosis* (Fig. 3c). Similar results were obtained when GM-CSF-matured MDMs from healthy controls were tested in the presence of infliximab (Fig. 3d). These findings were specific to TNF-deficient GM-CSF-matured MDMs, as MDM generation in the presence of M-CSF and IL-4 resulted in normal ROS production (Extended Data Fig. 7a–c). Western blotting of whole-cell lysates showed that all NADPH oxidase subunits were normally expressed in the patients’ phagocytes (Extended Data Fig. 7d,e). No defect in ROS production was observed in neutrophils and monocytes from P1 and P2 after stimulation with PMA, suggesting that the defect was macrophage specific (Extended Data Fig. 7f). GM-CSF-matured MDMs differentiated in the presence of infliximab or adalimumab produced lower levels of ROS after exposure to BCG, heat-killed *M. tuberculosis* or PMA after pretreatment with IFNγ (Extended Data Fig. 8a–c). Mitochondrial ROS levels in response to heat-killed *M. tuberculosis* or PMA in GM-CSF-matured MDMs or AML cells were unaffected by infliximab treatment, suggesting a mechanism specific to the activity of the phagocytic NADPH oxidase complex (Extended Data Fig. 8d,e).

## TNF–TNFR1 signalling mediates ROS production

TNF signals through the two transmembrane receptors, TNFR1 and TNFR2, which are both expressed on macrophages but control different signalling pathways. We investigated the possible effects of TNF on ROS production by signalling through one or both TNF receptors, by generating isogenic knockout (KO) iPS cells. iPS-cell-derived macrophages terminally matured with GM-CSF and deficient for TNF (*TNF* KO) or TNFR1 (*TNFR1* KO) displayed a similar impairment in ROS production after stimulation with PMA and heat-killed *M. tuberculosis*. By contrast, no such impairment was observed for cells deficient for TNFR2 (*TNFR2* KO), which were able to sense soluble TNF, indicating a mechanism involving TNFR1 but not TNFR2. ROS production was abolished in iPS-cell-derived macrophages deficient for gp91^phox^ (*CYBB* KO) (Fig. 3e–h). The priming of iPS-cell-derived macrophages with exogenous TNF rescued the defective ROS production after PMA stimulation in *TNF*-KO but not in *TNFR1*-KO or *CYBB*-KO cells (Fig. 3i). These results suggest that TNF signalling through TNFR1 is essential for NADPH oxidase complex activation in GM-CSF-matured MDMs. In summary, our findings suggest that a lack of TNF signalling during monocyte-to-macrophage differentiation in the presence of GM-CSF impairs ROS production by macrophages.

## ROS production in TNF-deficient AML cells

**Fig. 4:**
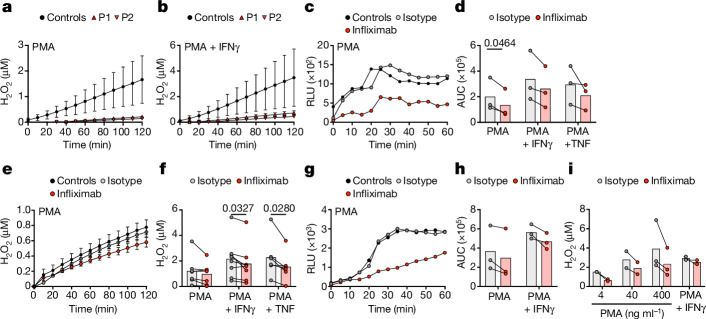
Loss of TNF signalling in AML cells and lung macrophages impairs NADPH oxidase activity. **a**,**b**, Extracellular H_2_O_2_ release in AML cells from healthy controls (*n* = 3) or patients (*n* = 2) after stimulation with PMA alone (**a**) or in combination with IFNγ (**b**). Data are mean ± s.d. and are representative of two independent experiments. **c**, The production of O_2_^−^ (RLU) by AML cells treated with infliximab or isotype control for 24 h before stimulation with PMA. Data representative of *n* = 3 biological replicates are shown. **d**, AUC analysis of O_2_^−^ production by AML cells (*n* = 3) in response to PMA with or without stimulation with IFNγ or TNF. **e**, Extracellular H_2_O_2_ release by AML cells treated as in **c**. Data are mean ± s.d. and are representative of six independent experiments. **f**, H_2_O_2_ release by AML cells (*n* = 6) after 30 min of stimulation treated as in **d**. **g**, The production of O_2_^−^ by lung macrophages treated as in **c**. Data representative of *n* = 3 biological replicates are shown. **h**, AUC analysis of O_2_^−^ production by lung macrophages (*n* = 3) treated as in **c** in response to PMA with or without IFNγ stimulation. **i**, Extracellular H_2_O_2_ release by lung macrophages (*n* = 2–3) after 30 min of treatment as in **c**. Cells were stimulated with IFNγ for 24 h or with PMA at the indicated doses at the start of the experiment. Significance was assessed using paired two-tailed *t-*tests (**d** and **f**).

Alveolar macrophages are thought to be among the first cells to be infected by *M. tuberculosis* in the alveolar space, and insufficient control of *M. tuberculosis* by these cells is thought to underlie TB^37,38^. We assessed the impact of TNF deficiency on ROS production in a macrophage type relevant to pulmonary TB infection, by generating AML cells from monocytes of healthy controls and both patients with TNF deficiency (Fig. 4a,b). AML cells from P1 and P2 released much less extracellular H_2_O_2_ in response to PMA or PMA plus IFNγ stimulation compared with those of healthy controls (Fig. 4a,b). Treatment of AML cells from healthy control individuals with infliximab decreased the levels of both O_2_^−^ and H_2_O_2_ produced in response to stimulation with PMA or PMA plus IFNγ, suggesting that TNF signalling is crucial for ROS production by AML cells in vitro (Fig. 4c–f). We also evaluated ROS production after treating human lung macrophages (composed of alveolar and interstitial macrophages) isolated from lung biopsy specimens with infliximab ex vivo (Fig. 4g–i). As observed for AML cells and GM-CSF-matured MDMs, the treatment of lung macrophages with infliximab decreased the amount of O_2_^−^ produced in response to stimulation with PMA or PMA plus IFNγ (Fig. 4g,h). Extracellular H_2_O_2_ levels were also lower for infliximab-treated lung macrophages that were treated with various doses of PMA or PMA plus IFNγ (Fig. 4i). Together, these findings obtained with AML cells in vitro and pulmonary macrophages ex vivo suggest that TNF signalling is important for ROS production by human alveolar macrophages in vivo.

## Response to *Listeria* and *M. tuberculosis*

**Fig. 5:**
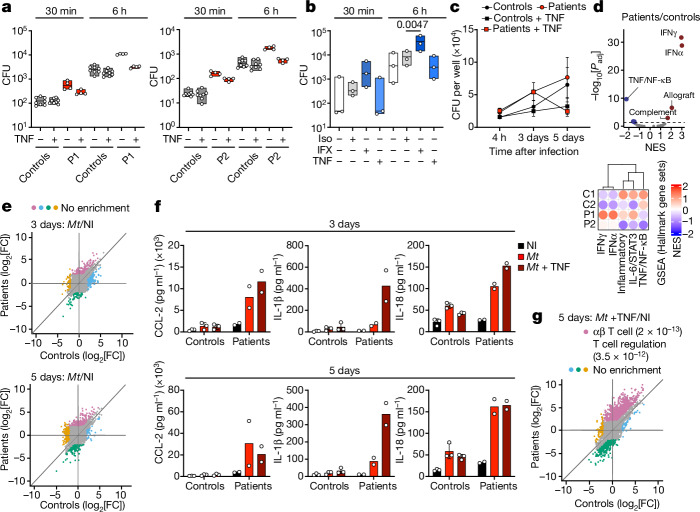
The role of TNF against *L. monocytogenes* and *M. tuberculosis.* **a**, GM-CSF-matured MDMs from two healthy controls and P1 or P2 were stimulated with TNF for 24 h or left unstimulated and were then infected with *L. monocytogenes*. The floating bars depict the minimum and maximum values and the median is indicated. *n* = 4 technical replicates. **b**, GM-CSF-matured MDMs from healthy controls were derived in the presence of infliximab (IFX), or the corresponding isotype (iso) control, or were stimulated with TNF and then infected with *L. monocytogenes* as in **a**. The floating bars show the minimum and maximum values, and the median of three independent experiments is indicated. **c**, AML cells from healthy controls (*n* = 3), and the two patients with TNF deficiency were either left unstimulated or were stimulated with TNF and infected with live *M. tuberculosis*. Data are mean ± s.d. **d**, GSEA of resting AML cells from patients (*n* = 2) and controls (*n* = 3) with the 50 hallmark gene set. Results are shown for selected immune-related gene sets. NES, normalized enrichment score. *P* values were estimated using fgsea gene set enrichment based on an adaptive multilevel split Monte Carlo scheme. **e**, The log_2_-transformed fold change (FC) difference in expression for genes that are differentially expressed between the AML cells of patients and healthy controls in response to *M. tuberculosis*. NI, not infected. **f**, Cytokine concentrations in the supernatants from the AML cells of controls (*n* = 3) and patients (*n* = 2) either non-infected or infected with *M. tuberculosis*, with or without TNF. Data are mean ± s.d. **g**, The log_2_-transformed fold change difference in mRNA levels after stimulation with TNF in combination with *M. tuberculosis* infection for transcripts differentially expressed between infected and non-infected conditions. Significance was assessed using two-tailed paired *t*-tests (**b**).

We next investigated whether cell-intrinsic immunity to intramacrophagic *L. monocytogenes* was altered in TNF-deficient myeloid mononuclear cells. GM-CSF-matured MDMs from both patients yielded more colony-forming units (CFUs) than those of the controls, attesting to higher rates of *L. monocytogenes* infection and/or multiplication (Fig. 5a). Stimulation of the patients’ cells with TNF led to a decrease in viable *L*. *monocytogenes* levels (Fig. 5a). Similar results were obtained with infliximab-treated healthy control GM-CSF-matured MDMs, which mimicked the phenotype of the patients’ cells. (Fig. 5b). Thus, TNF signalling in human GM-CSF-matured MDMs is crucial for the control of *L. monocytogenes* infection and/or replication. Finally, we infected AML cells generated from healthy controls and TNF-deficient patients with live *M. tuberculosis* in the presence or absence of TNF (Fig. 5c). The replication of *M. tuberculosis* was similar in AML cells from patients with TNF deficiency and healthy controls but was strongly decreased by TNF stimulation (Fig. 5c). These results suggest that TNF signalling is crucial for the control of *M. tuberculosis* in human AML cells. RNA-seq analysis of uninfected patient and control AML cells revealed a significant downregulation of genes associated with TNF signalling in the patients’ AML cells, as expected, whereas genes associated with inflammatory responses were significantly upregulated (Fig. 5d). GSEA of *M. tuberculosis*-infected AML cells detected no enrichment in patients relative to the controls for any pathway (Fig. 5e). However, the patients’ AML cells secreted larger amounts of proinflammatory cytokines in response to *M. tuberculosis* (Fig. 5f and Extended Data Tables 5 and 6). Supplementation with TNF in the context of *M. tuberculosis* infection enhanced the inflammatory response of the patients’ AML cells and increased the transcription of genes associated with T cell responses (Fig. 5f,g). These results indicate that TNF deficiency does not affect the transcriptional responses of AML cells to *M. tuberculosis* and suggest that other non-transcriptional mechanisms, such as impaired ROS production, underlie the susceptibility to *M. tuberculosis* of these patients.

## Discussion

Inherited TNF deficiency is a new genetic aetiology of recurrent pulmonary TB in adults. Our findings are consistent with both the susceptibility of TNF-deficient mice to *M. tuberculosis*^39,40^ and the reactivation of *M. tuberculosis* from latency often observed in patients on TNF blockade^33^. Both patients with TNF deficiency presented recurrent pulmonary TB with early relapse within 1 year of the end of treatment. The first episode was probably due to primary infection, whereas the second probably corresponded to short-term reactivation from latency. However, we cannot exclude the possibility of reinfection with the same or a different mycobacterial strain. The mechanism of disease probably involves the selective impairment of ROS production by alveolar macrophages in vivo, as suggested by our analysis of GM-CSF-matured MDMs and AML cells in vitro and lung macrophages ex vivo^2,13^. TNF deficiency does not appear to alter the constitutive expression of the NAPDH oxidase subunits in these macrophages derived in vitro, but it did reduce their priming of the phagocytic NADPH oxidase complex.

The impairment of ROS production in TNF-deficient phagocytes was narrower than that in patients with classic CGD, who display impaired ROS production in all types of phagocytes and are prone to multiple infections, including both BCG disease and TB^18,41^. Disruption of the respiratory burst in granulocytes and monocytes probably accounts for most infections other than BCG and TB in patients with CGD. The patients’ respiratory-burst phenotype, restricted to GM-CSF-matured macrophages (MDMs and AML cells) in vitro, was also narrower than that in patients with MSMD due to specific variants in *CYBB* selectively disrupting the respiratory burst in both GM-CSF- and M-CSF/IL-4-matured MDMs^20^, probably accounting for their clinical phenotype being restricted to TB, without BCG disease.

Human TNF deficiency underlying TB may have contributed to the rarity of LOF *TNF* variants in the populations tested. TB has probably killed more humans than any other single pathogen and has therefore exerted strong selective pressure on the human genome^42^. Purging operates much more rapidly for dominant than for recessive traits, but the intensity, prevalence and persistence of TB has purged variants in TB-susceptibility genes, such as *TYK2*, which underlie TB only in humans with biallelic lesions^42^. Nevertheless, the rarity of LOF variants may also result from other factors, including but not restricted to the impact of other infections. Moreover, not all populations have been tested, and LOF variants in *IFNAR1* and *IFNAR2*, which are exceedingly rare everywhere else, are common in the Pacific and Arctic regions, respectively^43,44^.

One of the two patients had listeriosis during pregnancy. The pregnancy may itself have contributed to the development of listeriosis^45^, but TNF deficiency may have also had an important role, as TNF-deficient mice are susceptible to *Listeria* and the rates of *Listeria* infection/replication were clearly high in the patients’ MDMs^46,47^. Moreover, *Listeria* is an intramacrophagic bacterium that can, albeit rarely, underlie clinical listeriosis in patients with CGD^48^. It is remarkable that these two patients managed to reach adulthood without experiencing any other major infectious diseases, despite living in Colombia, a country in which not only TB and listeriosis are endemic, but also various other intramacrophagic infections, including histoplasmosis, cryptococcosis, paracoccidioidomycosis, brucellosis, salmonellosis and leishmaniasis^49–53^. It is possible that the strong inflammatory responses of the patients’ AML cells observed in vitro might compensate for the loss of TNF activity.

It is also surprising that the two patients displayed normal clinical and biological inflammatory reactions throughout their lives, even during their episodes of TB and listeriosis. They had fever, high levels of inflammatory markers and they lost weight, and one patient even had septic shock during listeriosis; all of these features have long been considered to be due to TNF^54,55^. The patients even had well-formed tissue granulomas in response to *M. tuberculosis*, in contrast to TNF-deficient mice infected with *M. tuberculosis* or mice on anti-TNF treatment infected with BCG^40,56,57^. The two adult patients did not display any of the other known phenotypes of TNF-deficient mice, such as an impairment of the IgG antibody response after immunization^58,59^. Perhaps even more surprising, the patients displayed no new and unexpected phenotypes, even after more than two decades of TNF deficiency. Thus, human TNF seems to be largely redundant physiologically, but essential for the maturation and function of certain human macrophages, such as alveolar GM-CSF-dependent macrophages in particular, through its role in priming the NADPH oxidase complex and, therefore, in protective immunity to airborne *M. tuberculosis*.

## Methods

### Human participants

Both the patients and the relatives studied are alive and being followed up in Colombia. They were recruited by the Inborn Errors of Immunity group (formerly Primary Immunodeficiencies Group) in Medellín (Colombia). Written informed consent was obtained from the patients and relatives studied. This study was approved by the institutional ethics committees of the University of Antioquia (21-07-842), the Rockefeller University (JCA-0699) and INSERM (C10-07) and was performed in accordance with the local requirements of these institutions. Experiments on samples from human participants were conducted in the USA, France, Qatar and Colombia, in accordance with local regulations and with the approval of the institutional review boards of the corresponding institutions. Experiments on human lung tissue samples were approved by the regional institutional review board (Comité de Protection des Personnes Île de France VIII, Boulogne-Billancourt, France). Plasma samples from unrelated healthy individuals were collected at Sidra Medicine in accordance with a study protocol approved by the Clinical Research Ethics Board of Sidra Medicine, Qatar. Antibody profiles from selected, age-matched blood donors of diverse nationalities and individuals representative of the Arab general population were used for comparison (NCBI Sequence Read Archive: PRJNA685111 and PRJNA688708)^60^. Healthy volunteers for other studies were recruited in Colombia, France and the United States.

### Extended case reports for P1 and P2

P1 was born in 1994. She was vaccinated with BCG vaccine strain Pasteur at birth with no documented adverse reaction. She received other vaccines in accordance with the national immunization program of Colombia, with no adverse effects. At the age of 19 years, she had a cough for a duration of one month, with fever (40 °C), pleuritic pain and unexplained weight loss. Chest X rays and CT scans revealed multiple pulmonary micronodules and a nodular lesion in the right lung. A segmental lobectomy of the right upper lobe was performed and revealed the presence of well-constituted, paucibacillary granulomas, some of which were necrotic. Immunohistochemistry detected the presence of CD3, CD4 and CD8 T cells, and epithelioid cells with a few giant cells. Bacterial cultures of sputum, bronchoalveolar lavage fluid and lung tissue were positive for a strain of *M. tuberculosis* that was found to be rifampicin-susceptible on Xpert PCR analysis. QuantiFERON and TST results were positive. Complete remission was achieved after 12 months of oral treatment with isoniazid, rifampicin, pyrazinamide and ethambutol. At 22 years of age, P1 was hospitalized at 28 weeks of pregnancy for a vaginal and urinary tract infection. She went into septic shock shortly afterwards, and an emergency caesarean section was required to extract the fetus, due to clinical chorioamnionitis. A blood culture was positive for *L. monocytogenes*, leading to the initiation of 7 days of antimicrobial treatment with trimethoprim-sulfamethoxazole and ampicillin. The patient was discharged 10 days after admission. Then, 5 months later, P1 was again diagnosed with pulmonary TB. Xpert PCR on bronchoalveolar lavage fluid again confirmed infection with rifampicin-susceptible *M. tuberculosis*. At the age of 22 years, P1 was diagnosed with thymic hyperplasia, leading to thoracoscopy and complete thymectomy. Acid-fast bacillus (AFB) smear microscopy, Xpert PCR and cultures were negative for *M. tuberculosis*. A histological analysis of thymus tissue revealed mature adipose tissue, abundant thymic tissue with irregular islets but preserved architecture without signs of thymic carcinoma or thymoma. At the age of 23 years, P1 experienced a spontaneous abortion of unknown cause at 14 weeks of pregnancy. At the age of 27 years, she gave birth to a daughter. P1 is currently doing well without prophylactic treatment.

P2 was born in 1987 and is a first cousin of P1. He received all of the recommended vaccines according to the national vaccination schedule, including the live BCG vaccine (Pasteur strain), with no adverse effects. At the age of 18 years, he presented left pulmonary TB in the left lung with pleural effusion. P2 received 6 months of HRZE antimycobacterial treatment and responded well. However, he had a relapse eight months later and had to be hospitalized again. P2 was again diagnosed with TB, with positive QuantiFERON and TST results. He responded well to antimycobacterial treatment and is currently doing well off prophylactic treatment. Neither of the patients experienced any other severe infections caused by other bacteria, parasites or viruses. None of their relatives had a history of severe infectious diseases, including TB.

### Cell lines

HEK293T cells (ATCC, CRL-11268, verified by the manufacturer via STR profiling) were cultured in Dulbecco’s modified Eagle medium (DMEM) (Thermo Fisher Scientific, 11885084) supplemented with 10% fetal bovine serum (FBS). B cells from P1 and healthy controls were immortalized by infection with EBV and cultured in RPMI 1640 (Thermo Fisher Scientific, 11875093) supplemented with 10% FBS. HVS-T cells for P1 and healthy controls were generated with *H. saimiri* strain C488 for transformation or with the TERT transformation system. HVS-T cells were cultured in Panserin/RPMI 1640 (ratio 1:1) supplemented with 20% FBS, l-glutamine, gentamycin and 20 IU ml^−1^ human rIL-2 (Roche, 11147528001). Human iPS cells were maintained on mouse embryonic fibroblasts (MEFs) (Thermo Fisher Scientific, A34181) in knockout DMEM (Thermo Fisher Scientific, 10829-018) supplemented with 20% knockout serum replacement (Thermo Fisher Scientific, 10828-028), 2 mM l-glutamine (Thermo Fisher Scientific, 25030-024), 1% non-essential amino acids (Thermo Fisher Scientific, 11140-035), 1% penicillin–streptomycin (Thermo Fisher Scientific; 15140-122), 0.2% β-mercaptoethanol (Thermo Fisher Scientific, 31350-010) and 10 ng ml^−1^ basic fibroblast growth factor (bFGF, Peprotech, 100-18B). The cell lines were regularly tested and were found to be free of mycoplasma contamination.

### Genetics

Genomic DNA from P1, P2 and their relatives was used for WES. Exome capture was performed using the SureSelect Human All Exon V4+UTR and SureSelect Human All Exon V6 kits (Agilent Technologies). Paired-end sequencing was performed on the HiSeq 2000 sequencer (Illumina) generating 100 base reads. We used the Genome Analysis Software Kit (GATK) (v.3.4-46) best-practice pipeline to analyse our WES data. Reads were aligned with the human reference genome GRCh38 using BWA. PCR duplicates were removed using Picard tools v.3.1.1 (https://picard.sourceforge.net/). The GATK base quality-score recalibrator was used to correct sequencing artifacts. The GATK HaplotypeCaller v.4.1.4.1 was used to identify variant calls. Variants were annotated using SnpEff v.4.5 (https://snpeff.sourceforge.net/). Homozygosity rates were estimated from the patients’ genomic DNA, as previously described^61^. Parametric multipoint linkage analysis was performed on the WES data using MERLIN v.1.1.2, assuming AR inheritance with complete penetrance and a damaging allele frequency of 1 × 10^−4^. Allele frequencies were estimated for 72,817 SNPs with the gnomAD v.2.1.1 American population. Markers were clustered with an *r*^2^ threshold (--rsq parameter) of 0.4. The genetic variant of interest was confirmed by PCR amplification of the region surrounding *TNF* exons 2 and 3 from the gDNA (5′-AGCTGTTGAATGCCTGGAAGG-3′, and 5′-CTCAGCGAGTCCTTCTCACATTG-3′) followed by Sanger sequencing.

### Detection of copy-number variants

We searched for copy-number variants in patient samples by applying ExomeDepth^62^ and HMZDelFinder_opt^63^ to WES samples mapped onto the human reference genome GRCh38. For both analyses, we selected the 50 nearest neighbours based on coverage as controls. We discarded HMZDelFinder_opt low-confidence candidates with a *z* score of above −1.2. All of the results were checked manually by read-mapping with Alamut Visual Plus v.1.5. The results are provided in Extended Data Table 3.

### Phage immunoprecipitation sequencing

The reactivity of circulating antibodies against common pathogens in plasma samples from P1 and P2 and healthy controls was analysed by phage immunoprecipitation–sequencing (PhIP–seq) as previously described^64^. Pooled human plasma for IVIg (Privigen CSL Behring), human IgG-depleted serum (HPLASERGFA5ML, Molecular Innovations) and plasma samples from unrelated healthy adults were included as controls. PhIP–seq was performed with a modified version of the original VirScan phage library and data were processed as previously described^60,65^.

### Detection of autoantibodies

Recombinant *E. coli*-derived IFNγ (285-IF-100/CF, R&D Systems), IL-12 (10018-IL-020, R&D Systems), IL-23 (1290-IL-010, R&D Systems) and TNF (300-01A, Peprotech) were first biotinylated with EZ-Link Sulfo-NHS-LC-Biotin (A39257, Thermo Fisher Scientific) according to the manufacturer’s instructions with a biotin-to-protein molar ratio of 1:12. The detection reagent contained a secondary antibody (Alexa Fluor 647 goat anti-human IgG (A21445, Thermo Fisher Scientific)) diluted in Rexxip F (P0004825, Gyros Protein Technologies; 1:500 dilution of the 2 mg ml^−1^ stock to yield a final concentration of 4 mg ml^−1^). PBS supplemented with 0.01% Tween-20 (0.01% PBS-T) and Gyros wash buffer (P0020087, Gyros Protein Technologies) were prepared according to the manufacturer’s instructions. Plasma samples were diluted 1:100 in 0.01% PBS-T and tested with the Bioaffy 1000 CD (P0004253, Gyros Protein Technologies) and a Gyrolab xPand (P0020520, Gyros Protein Technologies).

The blocking activity of anti-IFNα2, anti-IFNβ and anti-IFNω autoantibodies was determined using a reporter luciferase activity. In brief, HEK293T cells were transfected with a plasmid containing the Firefly luciferase gene under the control of the human ISRE promoter in the pGL4.45 backbone, and a plasmid constitutively expressing *Renilla* luciferase for normalization (pRL-SV40). Cells in DMEM supplemented with 2% FBS and 10% healthy control or patient serum/plasma (after inactivation at 56 °C, for 20 min) were either left unstimulated or were stimulated with 100 ng ml^−1^ IFNα2, IFNβ or IFNω at a concentration of 10 ng ml^−1^ or 100 pg ml^−1^ for 16 h at 37 °C. Cells were lysed for 20 min at room temperature and luciferase levels were measured using the Dual-Luciferase Reporter 1000 assay system (E1980 Promega).

### Site-directed mutagenesis, transient and stable transfection

The pCMV3-SP-N-Myc-TNF vector was purchased from SinoBiological (HG10602-NM) and modified to remove the Myc tag sequence. Mutagenesis was performed with appropriate primers using the QuikChange II XL Site-Directed Mutagenesis Kit (Agilent Technologies, 200521). HEK293T cells were transiently transfected in the presence of Lipofectamine 2000 (Thermo Fisher Scientific, 11668019). For stable transfection, WT and mutant *TNF* sequences were inserted into the pTRIP-CMV-Puro-2A vector (Addgene, 102611). For lentivirus production, HEK293T cells were transfected with 0.2 µg pCMV-VSV-G (Addgene, 8454), 0.2 µg pHXB2 (NIH-AIDS Reagent Program; 1069), 1 µg psPAX2 (Addgene plasmid 12260) and 1.6 µg pTRIP-CMV-Puro-2A in the presence of Lipofectamine 2000. After transfection for 6 h, the medium was replaced with 3 ml DMEM supplemented with 10% FBS. The viral supernatant was collected 60 h later and concentrated with a Lenti-X Concentrator (Takara Bio, 631232). Concentrated lentivirus preparations were used for transduction in the presence of 10 µg ml^−1^ protamine sulfate (Merck, P3369).

### Western blotting

Cell lines and primary cells were lysed with modified radioimmunoprecipitation assay (RIPA) buffer (25 mM Tris–HCl pH 7.4, 150 mM NaCl, 1% NP-40 and 1 mM EDTA) supplemented with cOmplete ULTRA protease inhibitor cocktail tablets (Merck, 5892970001) and PhosSTOP tablets for phosphatase inhibition (Merck, 4906837001), 0.1 mM dithiothreitol (Thermo Fisher Scientific, 20290) and 1 mM PMSF (Merck, 10837091001). Protein samples were subjected to electrophoresis in 10–20% Criterion Tris-HCl Protein Gels (Bio-Rad, 3450043) or 4–20% TGX precast gels (Bio-Rad, 4561094), and the resulting bands were transferred onto Immobilon-P PVDF membranes (Millipore, IPVH00010). Membranes were probed with antibodies directed against TNF (C terminus, Abcam ab1793; and N terminus, Aviva Systems Biology, ARP80342_P050, both 1:1,000), GAPDH (Santa Cruz, sc-47724 and sc-25778 both 1:3,000), gp91^phox^ (Santa Cruz, sc-130543, 1:300), p67^phox^ (Merck, 07-002, 1:2,000), p47^phox^ (Merck, 07-001, 1:5,000), p40^phox^ (Merck, 07-503, 1:1,000), p22^phox^ (Santa Cruz Biotechnology, sc-130550, 1:2,000) and EROS (Atlas Antibodies, HPA045696, 1:1,250) followed by incubation with secondary HRP-coupled anti-mouse (GE Healthcare, NXA931, 1:5,000) or anti-rabbit (GE Healthcare, NA934V, 1:5,000) antibodies. Images were analysed using Image Lab v.5.1 (Bio-Rad). Uncropped western blots are provided in Supplementary Fig. 1.

### Deep immunophenotyping of leukocytes using spectral flow cytometry

Blood leukocyte subsets were phenotyped as previously described^28^. In brief, freshly thawed PBMCs were stained with LIVE/DEAD Fixable Blue (Thermo Fisher Scientific, L23105, 1:800 in PBS) and blocked by incubation with FcR Blocking Reagent (Miltenyi Biotec, 1:25) on ice for 15 min. The cells were washed and surface stained with the following reagents on ice for 30 min: Brilliant Stain Buffer Plus (BD Biosciences, 566385, 1:5), anti-γδTCR-BUV661 (BD Biosciences, 750019, 11F2, 1:50), anti-CXCR3-BV750 (BD Biosciences, 746895, 1C6, 1:20) and anti-CCR4-BUV615 (BD Biosciences, 613000, 1G1, 1:20) antibodies. Cells were then washed and surface stained by incubation with the following reagents on ice for 30 min: anti-CD141-BB515 (BD Biosciences, 565084, 1A4, 1:40), anti-CD57-FITC (BD Biosciences, 347393, HNK-1, 3:250), anti-Vδ2-PerCP (BioLegend, 331410, B6, 3:500), anti-Vα7.2-PerCP-Cy5.5 (BioLegend, 351710, 3C10, 1:40), anti-Vδ1-PerCP-Vio700 (Miltenyi Biotec, 130-120-441, REA173, 1:100), anti-CD14-Spark Blue 550 (BioLegend, 367148, 63D3, 1:40), anti-CD1c-Alexa Fluor 647 (BioLegend, 331510, L161, 1:50), anti-CD38-APC-Fire 810 (BioLegend, 356644, HB-7, 3:100), anti-CD27-APC H7 (BD Biosciences, 560222, M-T271, 1:50), anti-CD127-APC-R700 (BD Biosciences, 565185, HIL-7R-M21, 1:50), anti-CD19 Spark NIR 685 (BioLegend, 302270, HIB19, 3:250), anti-CD45RA-BUV395 (BD Biosciences, 740315, 5H9, 3:250), anti-CD16-BUV496 (BD Biosciences, 612944, 3G8, 3:500), anti-CD11b-BUV563 (BD Biosciences, 741357, ICRF44, 1:100), anti-CD56-BUV737 (BD Biosciences, 612767, NCAM16.2, 3:250), anti-CD4-cFluor YG568 (Cytek, R7-20042, SK3, 3:250), anti-CD8-BUV805 (BD Biosciences, 612889, SK1, 3:250), MR1 tetramer-BV421 (NIH Tetramer Core Facility, 1:100), anti-CD11c-BV480 (BD Biosciences, 566135, B-ly6, 1:40), anti-CD45-BV510 (BD Biosciences, 563204, HI30, 3:250), anti-CD33-BV570 (BioLegend, 303417, WM53, 3:250), anti-iNKT-BV605 (BD Biosciences, 743999, 6B11, 1:25), anti-CD161-BV650 (BD Biosciences, 563864, DX12, 1:25), anti-CCR6-BV711 (BioLegend, 353436, G034E3, 3:250), anti-CCR7-BV785 (BioLegend, 353230, G043H7, 1:40), anti-CD3-Pacific Blue (BioLegend, 344824, SK7, 3:250), anti-CD20-Pacific Orange (Thermo Fisher Scientific, MHCD2030, HI47, 1:50), anti-CD123-Super Bright 436 (Thermo Fisher Scientific, 62-1239-42, 6H6, 1:40), anti-Vβ11-PE (Miltenyi Biotec, 130-123-561, REA559, 3:500), anti-CD24-PE-Alexa Fluor 610 (Thermo Fisher Scientific, MHCD2422, SN3, 1:25), anti-CD25-PE-Alexa Fluor 700 (Thermo Fisher Scientific, MHCD2524, 3G10, 1:25), anti-CRTH2-biotin (Thermo Fisher Scientific, 13-2949-82, BM16, 1:50), anti-CD209-PE-Cy7 (BioLegend, 330114, 9E9A8, 1:25), anti-CD117-PE-Dazzle 594 (BioLegend, 313226, 104D2, 3:250), anti-HLA-DR-PE-Fire 810 (BioLegend, L243, 1:50) and anti-CD66b-APC (eBioscience, 1305118, G10F5 1:50) antibodies. The cells were then washed and incubated with streptavidin-PE-Cy5 (BioLegend, 405205, 1:3,000) on ice for 30 min. The cells were washed again, fixed by incubation in 1% PFA/PBS, washed again and acquired on the Aurora cytometer using SpectroFlo v.3.0 (Cytek). Subsets were manually gated using FlowJo v.10, and the results were visualized using R v.4.

### Whole-blood stimulation for cytokine secretion

Whole-blood samples were collected into heparin-containing collection tubes. Blood samples were diluted 1:2 in RPMI 1640 and incubated with IFNγ (Imukin, Boehringer Ingelheim), IL-12 (20 ng ml^−1^, R&D Systems, 219-IL), IL-23 (100 ng ml^−1^, R&D Systems, 1290-IL), live *Mycobacterium bovis* BCG Pasteur substrain at a multiplicity of infection (MOI) of 20 or PMA (40 ng ml^−1^, Sigma-Aldrich, P8139) and ionomycin (10 μM, Sigma-Aldrich, I9657). The supernatants were collected after 48 h and analysed using the LEGENDplex Human Inflammation Panel 1 (BioLegend, 740809).

### PBMC stimulation assay with BCG and IL-23

For PBMC stimulation, 2 × 10^5^ PBMCs were plated in 96-well, round-bottomed plates containing RPMI 1640 supplemented with 10% human serum and stimulated for 48 h with 2.5 ng ml^−1^ IL-1β (R&D Systems, 201-LB), 50 ng ml^−1^ IL-12 (R&D Systems, 219-IL), 100 ng ml^−1^ IL-23 (R&D Systems, 1290-IL) and 0.1 v/v% BCG. PMA and ionomycin were added for the last 24 h. The supernatants were subjected to LEGENDplex multiplex ELISA with Human Inflammation Panel 1.

### PBMC BCG stimulation assay

Freshly thawed PBMCs were dispensed into a 96-well round-bottomed plate at a density of 3 × 10^5^ cells per well, in 200 μl per well lymphocyte medium. Cells were stimulated with live BCG at an MOI of 1, IL-12 (5 ng ml^−1^, R&D Systems, 219-IL) or IL-23 (100 ng ml^−1^, R&D Systems, 1290-IL). After 40 h of stimulation, GolgiPlug (BD Biosciences, 555029, 1:1,000) was added. The supernatants were collected after 8 h and evaluated with LEGENDplex Human Inflammation Panel 1. Cells were stained by incubation with Zombie NIR dye (BioLegend, 1:2,000) at room temperature for 15 min, and were then incubated on ice for 30 min with FcR blocking reagent (Miltenyi Biotec, 130-059-901, 1:50), 5-OP-RU-loaded MR1 tetramer-BV421 (NIH Tetramer Core Facility, 1:200), anti-CD3-V450 (BD Biosciences, 560365, UCHT1, 1:450), anti-CD4-BUV563 (BD Biosciences, 612912, SK3, 1:450), anti-CD8-BUV737 (BD Biosciences, 612754, SK1, 1:450), anti-CD20-BV785 (BioLegend, 302356, 2H7, 1:150), anti-CD56-BV605 (BioLegend, 362538, 5.1H11, 1:50), anti-γδTCR-Alexa Fluor 647 (BioLegend, 331214, B1, 1:50), anti-Vδ1-FITC (Miltenyi Biotec, 130-118-362, REA173, 1:150), anti-Vδ2-APC-Fire 750 (BioLegend, 331420, B6, 1:1350), anti-Vα7.2-Alexa Fluor 700 (BioLegend, 351728, 3C10, 1:50), anti-iNKT-BV480 (BD Biosciences, 746788, 6B11, 1:50) and anti-Vβ11-APC (Miltenyi Biotec, 130-125-529, REA559, 1:150) antibodies. Cells were fixed by incubation with 2% PFA/PBS on ice for 15 min, and were then permeabilized/stained by incubation overnight at −20 °C in the perm buffer from the True-Nuclear Transcription Factor Buffer Set (BioLegend, 424401) with an intracellular cytokine panel: FcR blocking reagent, anti-IFNγ-BV711 (BioLegend, 502540, 4S.B3, 1:450), anti-TNF-BV510 (BioLegend, 502950, MAb11, 1:150), anti-IL-17A-PerCP-Cy5.5 (BioLegend, 512314, BL168, 1:1350), anti-T-bet-PE-Cy7 (BioLegend, 644823, 4B10, 1:1350) and anti-RORγT-PE (BD Biosciences, 563081, Q21-559, 1:50) antibodies. Cells were acquired on the Aurora cytometer (Cytek). Data were manually gated using FlowJo as previously described^10,27^ and then imported into R for further analysis. Cellular composition was visualized with uniform manifold approximation and projection based on the expression levels of CD3, CD4, CD8, CD20, CD56, γδTCR, Vδ1, Vδ2, Vα7.2, MR1, T-bet and RORγT, with the data downsampled to 10,000 cells per sample.

### scRNA-seq analysis of leukocytes

Cryopreserved PBMCs from P1 and P2, one Colombian adult control individual and two patients with CYBB deficiency (one with MSMD and one with CGD) were analysed using scRNA-seq as previously described^24^. Thawed cells were washed with medium and filtered with a 70-µm-mesh MACS SmartStrainer (Miltenyi Biotec, 130-098-462). Cells were then washed three times with PBS plus 0.5% FBS and were finally filtered again with a 40 µm cell strainer (Corning, 352340) before capture on the 10x Genomics Chromium chip. Libraries were prepared with the Chromium Single Cell 3’ Reagent Kit (v3 Chemistry) and sequenced on the Illumina NovaSeq 6000 sequencer (S1 flowcell). Sequences were preprocessed with CellRanger v.6 on the 10x Genomics Cloud Analysis platform (https://www.10xgenomics.com/products/cloud-analysis). Approximately 11,000 to 16,000 cells were captured per sample, with a mean of at least 24,000 reads per cell.

The data generated in this study were analysed together with historical controls from the laboratory, two previously reported adult controls and two patients with IRF1 deficiency^7^, and publicly available control PBMC datasets downloaded from the 10x Genomics web portal (https://support.10xgenomics.com/single-cell-gene-expression/datasets). Manually curated datasets were integrated with Harmony (v.3.8)^66^. Two runs of sequential graph-based clustering were performed. The second-round clustering focused on memory and effector T and NK cells to achieve cellular subset separation at the highest possible resolution. Clusters were manually identified with the aid of the SingleR v.2.60 pipeline^67^ guided by MonacoImmuneData^68^. The TotalSeq datasets from 10x were also used to determine the identity of each cluster. Principal component analysis was conducted on count data normalized through variance-stabilizing transformation (VST). Pseudobulk differential expression analysis was conducted using DESeq2 (v.1.40.2)^69^, excluding all public datasets. GSEA was conducted with the fgsea package v.1.30.0 by projecting the fold-change ranking onto the following MSigDB genesets (http://www.gsea-msigdb.org/gsea/msigdb/): H (Hallmark), C2 CP (Curated canonical pathways), C3 (Regulatory targets) and C5 (Gene ontologies). Intercellular communication analysis was performed using CellChat (v.1.5)^70^. All analyses were performed in R v.4 (http://www.R-project.org/)^71^.

### Differentiation of MDMs

CD14^+^ cells were isolated from PBMCs with CD14 MicroBeads (Miltenyi Biotec, 130-050-201). GM-CSF-matured MDMs were generated with M1-Macrophage Generation Medium XF (PromoCell, C-28055). For the generation of MDMs in the presence of M-CSF + IL-4, CD14^+^ cells were incubated for 7 days in RPMI + 10% FCS supplemented with M-CSF (R&D Systems, 216-MC), and were then allowed to differentiate in the presence of M-CSF + IL-4 (R&D Systems, 204-IL). The medium was replaced every 2 to 3 days.

### Isolation of human lung macrophages

Lung tissue samples were obtained from patients undergoing surgical resection for suspected lung carcinoma without previous chemotherapy (*n* = 5), chronic aspergillosis in a context of bronchiectasis (*n* = 1) or explantation in the context of chronic respiratory insufficiency in a context of pulmonary amyloidosis (*n* = 1). A lung sample taken from a healthy area and considered to be surgical waste was dissected free of the pleura, visible airways and blood vessels^72^.

Lung macrophages were isolated using the adhesion method^73^. Fluid collected from the washing of minced peripheral lung tissues was centrifuged (2,000 rpm for 10 min). The cell pellet was resuspended in RPMI medium with 10% FCS, 2 mM l-glutamine and antibiotics. The resuspended viable cells were then dispensed into 96-well plates. The plates were incubated for at least 1.5 h at 37 °C and non-adherent cells were removed by gentle washing. The adherent cells (approximately 3 × 10^4^ cells per well) were >95% pure macrophages, as determined by May–Grünwald–Giemsa staining and CD68 immunocytochemistry. The day after isolation, macrophages were washed twice, and 100 µl RPMI medium supplemented with 1% FBS was added to each well.

### Differentiation of AML cells

Monocytes were isolated from patients or healthy, unrelated controls and were differentiated into AML cells as previously described^2,13,74^. Monocytes were isolated from PBMCs using a classical monocyte isolation kit (Miltenyi Biotec, 130-117-337) and cultured in RPMI 1640 containing 10% human serum (H4522, Merck), 80 µg ml^−1^ poractant alfa (Curosurf, Chiesi) or 100 µg ml^−1^ Infasurf (ONY Biotec), 10 ng ml^−1^ GM-CSF (BioLegend, 572914), 5 ng ml^−1^ TGF-β (BioLegend, 781804) and 5 ng ml^−1^ IL-10 (BD, 554611) for 6 days. The surfactant and cytokines were replenished every other day.

### ROS production assays

ROS production by neutrophils and monocytes was analysed by subjecting whole blood to red-blood-cell lysis and then incubating the lysate with dihydrorhodamine 123 (Thermo Fisher Scientific, D23806) in the presence or absence of PMA (400 ng ml^−1^). Monocytes were labelled with a CD14 Pacific-Blue-conjugated antibody (BD, M5E2, 558121, 1:50). The analysis was performed on the LSRFortessa Cell Analyzer (BD). Superoxide production by MDMs, AMLs, lung macrophages and EBV-B cells was evaluated using the Superoxide Anion Assay Kit (Sigma-Aldrich, CS1000) according to the manufacturer’s instructions. In brief, 3 × 10^4^ MDMs, AML cells or lung macrophages, or 1 × 10^5^ EBV-B cells per well were plated in a 96-well plate in the presence or absence of superoxide dismutase and stimulated with PMA (400 ng ml^−1^) or serum-opsonized heat-killed *M. tuberculosis* (InvivoGen, tlrl-hkmt-1). Luminescence was recorded on the Victor X4 plate reader. H_2_O_2_ production by MDMs, AML cells, lung macrophages and EBV-B cells was assessed using Amplex Red hydrogen peroxide (Thermo Fisher Scientific, A22188) in Krebs–Ringer phosphate buffer. In brief, 3 × 10^4^ cells per well were plated in 96-well flat-bottomed plates, stimulated for 24 h with 100 IU ml^−1^ IFNγ or 20 ng ml^−1^ TNF (R&D Systems, 210-TA). H_2_O_2_ release was measured after stimulation with PMA or serum-opsonized heat-killed *M. tuberculosis* on the Victor X4 (Perkin Elmer) plate reader.

### Immunofluorescence microscopy

Superoxide production by mitochondria was analysed using confocal microscopy with the MitoSOX Red Mitochondrial Superoxide Indicator (Thermo Fisher Scientific, M36008). We plated 4 × 10^5^ cells GM-CSF-matured MDMs and AML cells from healthy controls on coverslips (80826, iBidi) and treated them with infliximab (5 µg ml^−1^) or isotype control (5 µg ml^−1^) for 48 h. Cells were stimulated with PMA (400 ng ml^−1^), 0.5% DMSO or heat-killed *M. tuberculosis* (330 µg ml^−1^) for 4 h and were then incubated with 800 nM MitoSOX and 150 nM MitoTracker Green FM (Thermo Fisher Scientific, M7514) in HBSS buffer for 30 min at 37 °C. Cells were washed with HBSS and stained by incubation for 5 min with 1 µg ml^−1^ Hoechst 33342 (Thermo Fisher Scientific, 62249). Specific fluorescence was acquired with a Leica TCS SP8 STED (Leica) confocal laser-scanning microscope (×63 oil immersion lens).

### Generation of KO iPS cell lines

iPS cell KO lines were generated by the Stem Cell Research facility at MSKCC. CRISPR sgRNAs targeting the gene of interest were designed using the algorithm available online (https://www.benchling.com/crispr/). The target sequences were inserted into the pX330-U6-Chimeric_BB-CBh-hSpCas9 vector (Addgene, 42230) for the generation of specific gene-targeting constructs. Variants were introduced into the parental C12 iPS cell line by electroporating single-iPS-cell suspensions (1 × 10^6^ cells per reaction) with 4 µg sgRNA-construct plasmid in Nucleofector human stem cell solution (Lonza, VPH-5012). The electroporated cells were cultured for 4 days in feeder-cell-free conditions and were then passaged at low density to obtain single-cell colonies. Individual colonies were picked 10 days later, expanded and analysed using PCR. The lines obtained were karyotyped to check for the absence of chromosomal abnormalities.

### Generation of iPS-cell-derived macrophages

We differentiated macrophages from human iPS cells as previously described^75,76^. In brief, iPS cells were maintained for three days in iPS cell medium (with 10 ng ml^−1^ bFGF) and then 4 days in iPS cell medium only. On day 7, iPS cell colonies were transferred to low-adhesion plates (Thermo Fisher Scientific, 657185) in the presence of 10 μM ROCK Inhibitor (Sigma-Aldrich, Y0503). After 6 days of incubation, embryoid bodies were transferred to hematopoietic differentiation medium (APEL 2 medium (Stem Cell Tech, 05270) supplemented with 5% protein-free hybridoma (Thermo Fisher Scientific, 12040077), 1% penicillin–streptomycin, 25 ng ml^−1^ IL-3 (Peprotech, 200-03) and 50 ng ml^−1^ M-CSF (Peprotech, 300-25). From day 25 of differentiation onwards, cells in suspension were carefully collected, cultured for 6–10 days in RPMI medium + 10% FBS and 100 ng ml^−1^ GM-CSF (Peprotech, 300-03).

### *L. monocytogenes* infection

GM-CSF-matured macrophages were treated for 48 h with IFNγ (10^3^ IU ml^−1^) and TNF (50 ng ml^−1^). Infliximab or isotype control (both 5 µg ml^−1^) was added on days 1 and 7 of macrophage differentiation. Cells were infected with *L. monocytogenes* (MOI of 20 for 1 h at 37 °C). They were then washed twice and incubated with gentamicin (25 µg ml^−1^) for 30 min, 3 h or 6 h. The cells were washed and lysed with 0.05% Triton X-100 in PBS and serial dilutions were plated on BHI agar and incubated overnight.

### Infection of macrophages with *M. tuberculosis*

*M. tuberculosis* strain Erdman was used for the infection of AML cells. *M. tuberculosis* was grown to exponential growth phase in 7H9 Middlebrook medium supplemented with 0.5% glycerol, 0.05% Tween-80 and 10% oleic acid-albumin-dextrose-catalase (OADC), with shaking at 200 rpm. A single-cell mycobacterial suspension was prepared by washing bacteria collected by centrifugation twice with phosphate-buffered saline supplemented with 0.05% Tween-80 (PBST_80_) and then centrifuging at 200*g* for 10 min. AML cells were infected at an MOI of 5, calculated from the optical density at 600 nm (OD_600_) of the final suspension, considering an OD_600_ of 1.0 to equate to approximately 5 × 10^8^ CFU per ml. After 4 h, the cells were washed once with RPMI supplemented with 10% human serum and cultured for up to 5 days.

### Cytokine production and intracellular growth of *M. tuberculosis* in AML cells

The supernatant of *M. tuberculosis*-infected AML cells was passed through a filter with 0.22 µm pores twice and was then stored at −80 °C. Cytokine levels were quantified using LEGENDplex Human Inflammation Panel 1 (BioLegend, 740809) and LEGENDplex Human Cytokine Panel 2 (BioLegend, 741378). For the isolation of *M. tuberculosis* from infected AML cells, 0.01% Triton X-100 in sterile water was added to the wells and the plates were then incubated at room temperature for 20 min. The culture was then passed up and down in a pipette to ensure AML cell lysis. Cell lysates were diluted with PBST_80_ and cultured on 7H10 Middlebrook agar supplemented with 0.5% glycerol and 10% OADC for 2 weeks at 37 °C. RNA was prepared by washing cell monolayers with PBS and then extracting the RNA in TRIzol.

### RNA-seq analysis

We used the NovaSeq S1 platform (single-end, 100 bp reads), aiming to obtain 20–30 million reads per sample. The RNA-seq fastq raw data were inspected to ensure that they were of high quality and then mapped onto the human reference genome GRCh38 using STAR aligner (v.2.7)^77^. The aligned RNA-seq BAM files were used to quantify the gene-level read counts using featureCounts (v.1.6.0)^78^; these counts were then vst-normalized and log_2_-transformed using DESeq2 (v.1.40.2)^69^ to obtain values for the expression of all genes and all samples. The data were then analysed to identify the genes differentially expressed between the patients and the controls in baseline conditions without infection and at various timepoints (days 3 and 5) after stimulation (*M. tuberculosis*, *M. tuberculosis* + TNF). We used the fgsea package v.1.30.0 to perform GSEA for MSigDB hallmark gene sets and gene ontologies.

### Statistical analysis

All statistical analyses were performed using R v.4 (http://www.R-project.org/) and GraphPad Prism software v.9.5.0 (GraphPad). The statistical significance of quantitative differences between groups was assessed using Mann–Whitney *U*-tests or unpaired two-tailed Student’s *t*-tests. The statistical significance of differences between treated and untreated samples from the same donor was assessed using paired two-tailed Student’s *t*-tests. *P* values are indicated only for statistically significant differences.

### Reporting summary

Further information on research design is available in the Nature Portfolio Reporting Summary linked to this article.

## Online content

Any methods, additional references, Nature Portfolio reporting summaries, source data, extended data, supplementary information, acknowledgements, peer review information; details of author contributions and competing interests; and statements of data and code availability are available at 10.1038/s41586-024-07866-3.

## Supplementary information

Supplementary Fig. 1 Uncropped images from the western blots displayed in the figures indicated.

Reporting Summary

Supplementary Fig. 2 Gating strategy for spectral flow cytometry immunophenotyping.

## Figures and Tables

**Extended Data Table 1:**
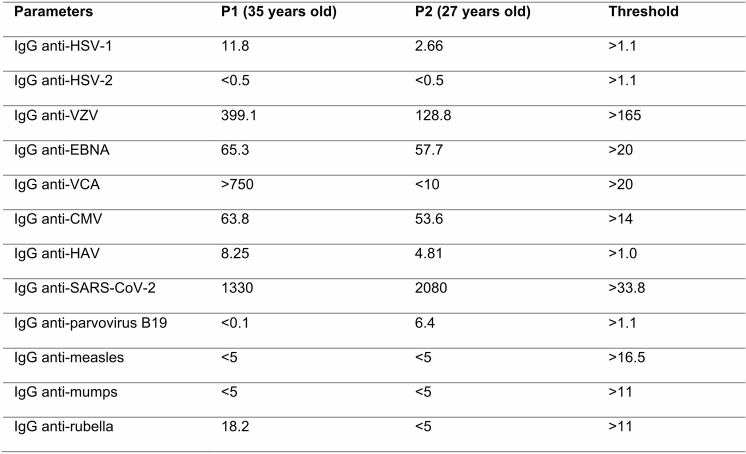
Serological results for antibodies against common viruses for the two patients

**Extended Data Table 2:**
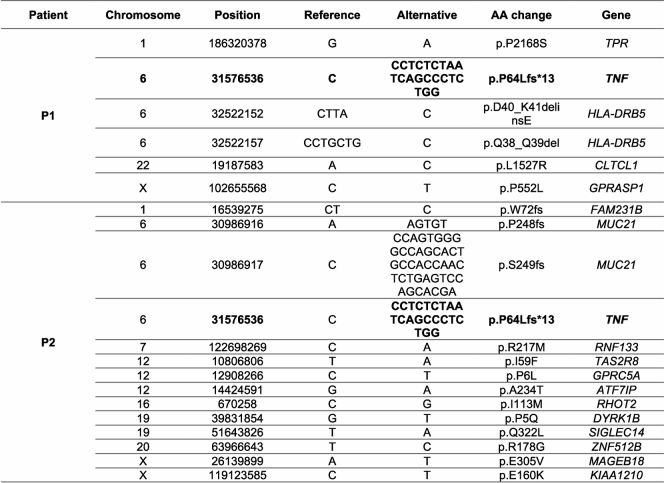
List of variants identified, by WES, in the homozygous or hemizygous state in the two TNF-deficient patients

For the female patient (P1) only homozygous variants are displayed (GRChr38). Variants were filtered such that those retained were non-synonymous and/or predicted to affect splice sites, to have a minor allele frequency <0.01 in any gnomAD v2.1.1 population and to have a CADD > MSC.

**Extended Data Table 3:**
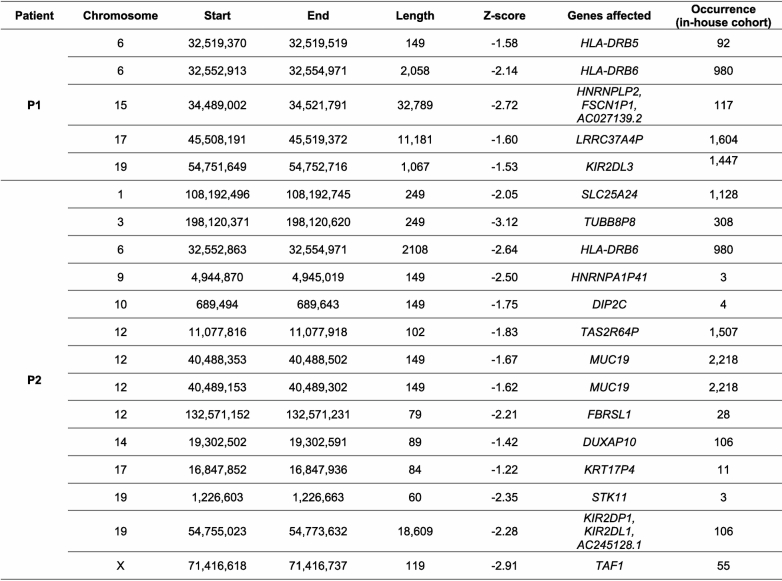
List of potential copy-number variants identified in the homozygous state in the two-TNF deficient patients with ExomeDepth and HMZDelFinder

**Extended Data Table 4:**
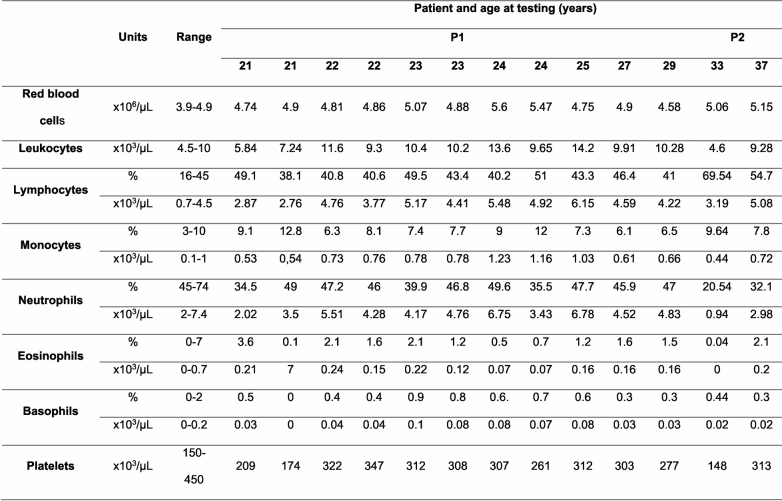
Whole-blood cell counts for the TNF-deficient patients at the ages indicated

**Extended Data Table 5:**
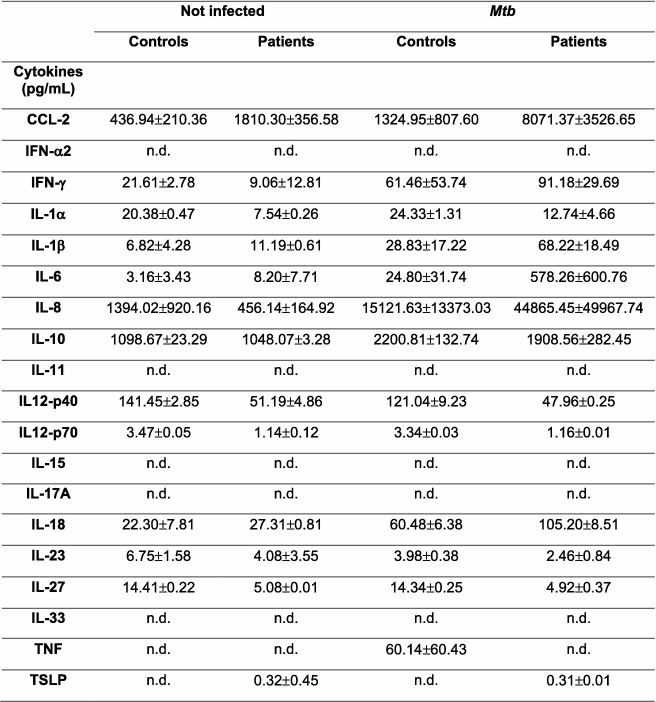
Levels of cytokines in the supernatants of cells from healthy controls (*n* = 3) and two TNF-deficient patients with and without infection with *Mtb*, 3 days post infection. The data shown are the mean ± s.d. n.d., not detectable

**Extended Data Table 6:**
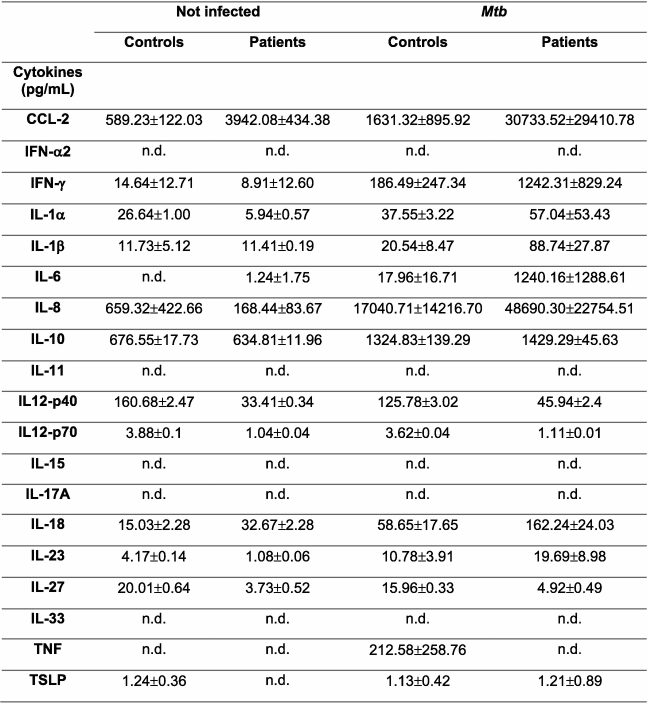
Levels of cytokines in the supernatants of cells from healthy controls (*n* = 3) and two TNF-deficient patients with and without infection with *Mtb*, 5 days post infection. The data shown are the mean ± s.d. n.d., not detectable

## Data Availability

All data supporting the findings of this study are available within the Article and its Supplementary Information. The gel source data are shown in Supplementary Fig. 1. Gating strategies for flow cytometry data are shown in Extended Data Fig. 3a and Supplementary Fig. 2. The RNA-seq and scRNA-seq data generated for this project are accessible from the NCBI SRA database under BioProject ID PRJNA1089511. scRNA-seq data from seven previously published adult controls can be found under BioProject IDs PRJNA818002 and PRJNA936917. The human reference genome GRCh38 is available under BioProject ID PRJNA3157. All other data and material supporting the findings of this study are available under a data transfer agreement from the corresponding authors on reasonable request.
